# Integrated proteomic and transcriptomic profiling identifies aberrant gene and protein expression in the sarcomere, mitochondrial complex I, and the extracellular matrix in Warmblood horses with myofibrillar myopathy

**DOI:** 10.1186/s12864-021-07758-0

**Published:** 2021-06-11

**Authors:** Zoë J. Williams, Deborah Velez-Irizarry, Keri Gardner, Stephanie J. Valberg

**Affiliations:** Large Animal Clinical Sciences, College of Veterinary Medicine, Michigan State University, 784 Wilson Road, East Lansing, MI 48824 USA

**Keywords:** Myofibrillar myopathy, Warmblood, Gluteal muscle, Proteomics, Transcriptomics, Z-disc

## Abstract

**Background:**

Myofibrillar myopathy in humans causes protein aggregation, degeneration, and weakness of skeletal muscle. In horses, myofibrillar myopathy is a late-onset disease of unknown origin characterized by poor performance, atrophy, myofibrillar disarray, and desmin aggregation in skeletal muscle. This study evaluated molecular and ultrastructural signatures of myofibrillar myopathy in Warmblood horses through gluteal muscle tandem-mass-tag quantitative proteomics (5 affected, 4 control), mRNA-sequencing (8 affected, 8 control), amalgamated gene ontology analyses, and immunofluorescent and electron microscopy.

**Results:**

We identified 93/1533 proteins and 47/27,690 genes that were significantly differentially expressed. The top significantly differentially expressed protein CSRP3 and three other differentially expressed proteins, including, PDLIM3, SYNPO2, and SYNPOL2, are integrally involved in Z-disc signaling, gene transcription and subsequently sarcomere integrity. Through immunofluorescent staining, both desmin aggregates and CSRP3 were localized to type 2A fibers. The highest differentially expressed gene *CHAC1*, whose protein product degrades glutathione, is associated with oxidative stress and apoptosis. Amalgamated transcriptomic and proteomic gene ontology analyses identified 3 enriched cellular locations; the sarcomere (Z-disc & I-band), mitochondrial complex I and the extracellular matrix which corresponded to ultrastructural Z-disc disruption and mitochondrial cristae alterations found with electron microscopy.

**Conclusions:**

A combined proteomic and transcriptomic analysis highlighted three enriched cellular locations that correspond with MFM ultrastructural pathology in Warmblood horses. Aberrant Z-disc mechano-signaling, impaired Z-disc stability, decreased mitochondrial complex I expression, and a pro-oxidative cellular environment are hypothesized to contribute to the development of myofibrillar myopathy in Warmblood horses. These molecular signatures may provide further insight into diagnostic biomarkers, treatments, and the underlying pathophysiology of MFM.

**Supplementary Information:**

The online version contains supplementary material available at 10.1186/s12864-021-07758-0.

## Background

Myofibrillar myopathy (MFM) is classically known as a late-onset protein aggregate myopathy in humans that can affect skeletal and cardiac muscle leading to muscle atrophy, weakness, respiratory compromise, and cardiomyopathy [1–3]. In humans, at least 8 genes, some containing more than 70 different mutations, cause MFM types 1–8 and an additional 8 genes are associated with MFM-like protein aggregate myopathies [3–6]. The cause of MFM in approximately 50% of human patients, however, remains unknown [6]. The variety of genes causing desmin aggregate myopathies and the heterogeneous clinical signs that arise over a wide range of ages, suggest that the underlying basis for MFM is complex, influenced by both genetic and environmental factors [6, 7].

MFM has recently been described in adult horses of Arabian and Warmblood (WB) breeds [8, 9]. Adult WB horses are diagnosed with MFM at on average 11 years-of-age and show clinical signs of exercise intolerance, a reluctance to move forward under saddle, a mild lameness and mild to moderate muscle atrophy [9, 10]. Thus, MFM can severely impact a horse’s athletic career and even breeding potential. Paralleling MFM in humans, horses with MFM have myofilament disarray, Z-disc disruption, desmin aggregation, focal accumulation of granulofilamentous material and clusters of degenerate mitochondria in skeletal muscle [3, 8, 9, 11]. Despite the similar histopathologic findings, there has been no underlying monogenic cause identified in WB with MFM. Commercial testing for MFM is not currently recommended by the authors due to a lack of correlation between the variants evaluated in the genetic tests and a diagnosis of MFM by histopathology [12]. Sixteen candidate MFM genes found to be associated with MFM or MFM-like myopathies in humans have been examined in MFM WB horses and no significant coding variants were identified when compared to control WB and publicly available data [13]. While an underlying genetic cause is still be possible, current findings suggest that MFM in WB is likely a complex disease with strong environmental influences. The etiopathology of MFM in WB could share similarities with the 50% of human MFM cases that have an unknown –potentially complex– etiology.

Transgenic animal models have confirmed the pathologic impact of some genetic mutations that result in MFM in humans [5, 14–18]. However, tightly controlled laboratory environments, homogeneous genetic backgrounds, small animal size, and reduced life expectancy of laboratory animals make it difficult to assess important variables that may impact the expression of diseases in humans [19]. A naturally occurring model of canine or equine MFM would be beneficial to further evaluate the complex mechanisms causing myofibrillar disruption and protein aggregation [8, 9, 20].

The current knowledge base of underlying pathophysiologic mechanisms makes treatment options for MFM in humans and horses limited. Identifying new biomarkers through integrated proteomic, transcriptomic and metabolomic analyses could provide more targeted treatments for this complex disease [21–24]. Multi-omic approaches highlight key pathways and cellular responses that stretch beyond the predictive measures of genomic variation and causative mutations [22]. Transcriptomic and proteomic profiling was employed to delineate underlying pathophysiology of MFM in Arabian horses [25]. However, a combined analysis interweaving both transcriptomic and proteomic data to highlight disease pathways has yet to be implemented in either equine or human MFM.

Transcriptomic and proteomic profiling of gluteal muscle in endurance-trained Arabian MFM horses highlighted alterations in cysteine-based antioxidants and metabolic pathways linked to oxidative stress [25]. Arabian horses with MFM, however, have much greater stamina than MFM WB and different genetic backgrounds, therefore the underlying pathophysiology may have different molecular signatures between breeds. The variation in clinical presentation and severity of equine MFM between Arabians and Warmbloods suggests that they could be separate diseases or MFM could represents a complex interaction of multiple gene sets of low effect size that are influenced by environmental factors.

We hypothesized that biomarkers and unique molecular signatures of MFM WB could be elucidated by integrating proteomic and transcriptomic analyses. The objectives of our study were to 1) identify differentially expressed proteins (DEP) and their pathways in MFM WB muscle using proteomic analyses, 2) identify differentially expressed gene transcripts (DEG) and their pathways in gluteal muscle from MFM and control WB using mRNA-sequencing, and 3) integrate the data to identify overarching molecular signatures of MFM in WB and their correspondence to muscle ultrastructure.

## Results

### Proteomics

#### Technical replicates of endogenous control

A control sample was divided into two technical replicates; each was run in triplicate. There was a significant correlation in spectral quantification within runs (r = 1.00) and across runs (r = 0.98–1.00), indicating that internal assay validation of runs and technical replicates was achieved.

#### Differential expression

**Fig. 1:**
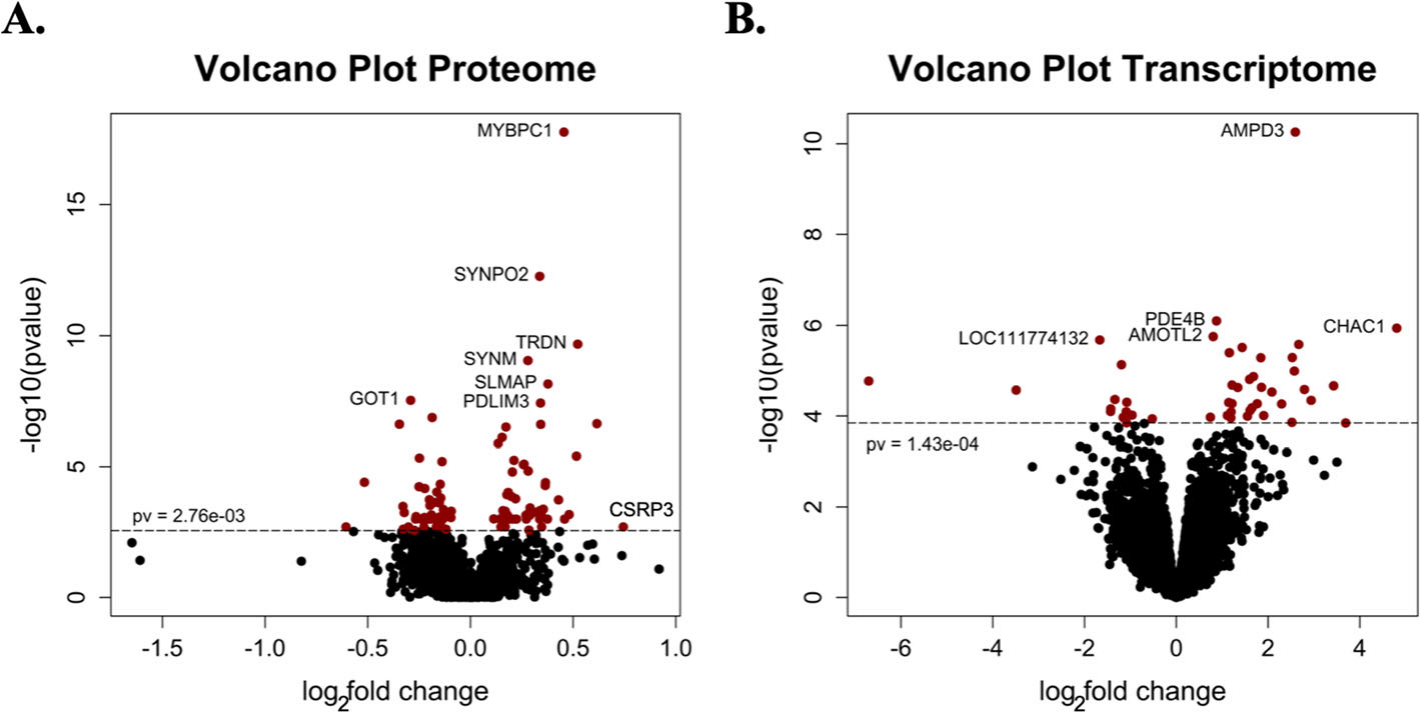
**A**. Protein expression according to the adjusted P value and the log_2_ fold change for 1533 proteins. Ninety-three of the proteins were significantly DE (*P* ≤ 0.0027) between MFM and control WB. **B**. Gene expression according to the adjusted P value and the log_2_ fold change for 14,366 genes. Forty-seven of the DE genes were significantly DE (*P* ≤ 0.0001) between MFM and control WB

There were 93 significantly DEP out of 1533 proteins identified in MFM versus control WB (*P* <  0.003, FDR ≤ 0.05) (Fig. 1A). Foutry-nine DEP had increased expression and 44 DEP had decreased. The protein with the highest log_2_ FC was a Z-disc protein CSRP3 (log_2_ FC 0.74) and the protein with the most negative log_2_ FC was D-dopachrome decarboxylase (DDT*,* log_2_ FC − 0.61). The 26 DEP with a log_2_ FC ≥ 0.30 generally had functions in the sarcomere and Z disc, mitochondria complex 1 and protein processing (Table 1). Four blood-borne proteins including fibrinogen and thrombospondin were also DEP.

**Table 1:** Significantly DE proteins with a log_2_ fold change of ≥0.30 in MFM WB compared to control WB

Cellular location/ process	Gene ID	Protein	Log_**2**_ FC	P adjusted	Function	Detected in transcriptomics
Sarcomere	CSRP3	Cysteine and glycine-rich protein 3	↑0.74	0.002	Z-disc regulator of myogenesis	No
	SMTNL1	Smoothelin-like protein 1	↑0.62	< 0.0001	Regulates contractile properties	Yes
	MYBPC1	Myosin-binding protein C, slow-type	↑0.46	< 0.0001	Myosin-muscle contraction, creatine kinase binding	Yes
	PDLIM3	PDZ and LIM domain protein 3	↑0.34	< 0.0001	Z-disc cytoskeletal organization, maintenance	Yes
	SYNPO2	Synaptopodin-2	↑0.34	< 0.0001	Z-disc cytoskeletal organization, maintenance	Yes
	TNNT1	Troponin T, slow skeletal muscle	↓0.33	0.0003	Thin filament contractility	Yes
	NEB	Nebulin	↓0.31	0.0005	Thin filament integrity	Yes
Cytoskeleton	EML1	Echinoderm microtubule-associated protein-like 1	↑0.35	0.002	microtubule cytoskeleton	Yes
Mitochondria	NDUFV3	NADH dehydrogenase [ubiquinone] flavoprotein 3	↑0.48	0.0007	complex 1 electron transfer	Yes
	MTND4	NADH-ubiquinone oxidoreductase chain 4	↓0.52	< 0.0001	complex 1 assembly and catalysis	No
	APOO	MICOS complex subunit	↓0.33	0.0024	maintenance of cristae	Yes
Protein processing	HNRNPA1	Heterogeneous nuclear ribonucleoprotein A1	↑0.43	0.0002	mRNA processing	Yes
	EEF2K	Eukaryotic elongation factor 2 kinase	↑0.37	0.001	Regulates protein synthesis	Yes
	UCHL1	Ubiquitin carboxyl-terminal hydrolase isozyme L1	↑0.34	0.001	Thiol protease- processing of ubiquinated proteins	Yes
	EIF3C	Eukaryotic translation initiation factor 3 subunit C	↑0.32	0.0006	mRNA processing	No
	BCAP31	B-cell receptor-associated protein 31	↑0.34	0.0005	Protein chaperone	Yes
	ASNA1	ATPase ASNA1	↓0.32	0.0006	Post-translational delivery proteins to ER	Yes
Cytoplasm	DDT	D-dopachrome decarboxylase	↑0.61	0.002	D-dopachrome to 5,6-dihydroxyindole	No
	CAT	Catalase	↓0.30	0.002	Antioxidant- (cytoplasm, mitochondria and peroxisomes)	Yes
Sarcoplasmic reticulum	TRDN	Triadin	↑0.52	< 0.0001	Calcium release complex	Yes
Sarcolemma	SLMAP	Sarcolemmal membrane-associated protein	↑0.38	< 0.0001	Unfolded protein binding	Yes
Extracellular matrix	CDH13	Cadherin-13	↑0.35	0.0004	Cell-cell adhesion	Yes
Extracellular	HP	Haptoglobin	↑0.52	< 0.0001	preproprotein for haptoglobin- binds hemoglobin	No
	FGB	Fibrinogen beta chain	↑0.46	0.001	Inflammation/blood clot	Yes
	FGG	Fibrinogen gamma chain	↑0.36	< 0.0001	Inflammation/blood clot	No
	FGA	Fibrinogen alpha chain	↑0.34	0.0006	Inflammation/blood clot	No
	APOA1	Apolipoprotein A-I	↓0.35	< 0.0001	Cholesterol transport	No

### Transcriptomics

#### mRNA reads and mapping

A sequencing depth of approximately 75.6 X per horse was achieved. An average of 56 ± 13.9 million reads per horse was filtered resulting in 76.4% of the filtered reads mapping to the equine genome, EquCab 3.0. Of those reads, 97.2% were unique and retained for downstream analysis. After filtering out genes with low read counts, 14,366 total genes were quantified (55.6% of the total raw reads and 51.9% of the total annotated genes) for DEG analysis between MFM and control WB (Additional File 1).

#### Differential expression

There were 47 significantly DEG out of 14,366 genes identified in MFM WB versus control WB with increased DEG for 34 transcripts and decreased for 13 (Fig. 1B). The log_2_ FC ranged from − 6.7 for hemoglobin subunit beta (*HBB,*) to 4.8 for glutathione specific gamma-glutamylcyclotransferase 1 (*CHAC1*). Eight of the 47 transcripts were novel transcripts unannotated in the current equine reference genome and 2 of the transcripts with locus identification were uncharacterized. Eleven of the 47 DEG (23%) had a log_2_ FC > 2 and are either transcription factors, involved in thiol-based glutathione degradation, thiol-based inhibition of ubiquitination, or erythrocyte energy metabolism (Table 2).

**Table 2:** Significantly DE annotated genes with a log_2_ FC > 2 in MFM WB compared to control WB

Function	Gene ID	Gene	log_**2**_ FC	P adjusted	Function	Detected in proteomics
Transcription factors	***CCR7***	C-C Motif Chemokine Receptor 7	↑3.4	0.02	Lymphocyte activation	no
	***NR4A2***	Nuclear receptor subfamily 4 group A member 2	↑2.9	0.031	Steroid-thyroid hormone-retinoid receptor	no
	***GADD45G***	Growth Arrest and DNA Damage Inducible Gamma	↑2.6	0.014	Response to environmental stresses	no
	***ATF3***	Cyclic AMP-dependent transcription factor ATF-3	↑2.5	0.05	Cellular stress response	no
	***CEBPD***	CCAAT/enhancer-binding protein delta	↑2.5	0.009	Immune and inflammatory responses, myostatin	no
Thiol-dependent	***CHAC1***	Glutathione-specific gamma-glutamylcyclotransferase 1	↑4.8	0.006	Glutathione degradation, apoptosis, Notch signaling	no
	***OTUD1***	OTU domain-containing protein 1	↑2.8	0.021	Thiol-dependent ubiquitin-specific protease activity	no
Immune response	***ADAMDEC1***	ADAM Like Decysin 1	↓3.5	0.021	Disintegrin metalloproteinase	no
Cell-cell interactions	***THBS1***	Thrombospondin	↑2.7	0.007	Blood clot formation, inhibits angiogenesis	no
Erythrocyte	***AMPD3***	AMP deaminase 3	↑2.6	< 0.0001	Deaminase activity (erythrocyte form)	no
	***HBB***	Hemoglobin subunit beta	↓6.7	0.019	Oxygen and iron binding	yes not DEP

#### Comparative differential protein and gene expression

There was low correlation between DEG and DEP. None of the 1229 identified gene IDs that were common to both the transcriptomic and proteomic datasets were significantly DE in both datasets. None of the DEG were expressed in the proteomic data. Only *HBB* from the transcriptomic data > 2 log_2_ FC was also present in the proteomic analysis, however it was not DE. Many of the 27 DE proteins with > 0.3 log_2_ FC were also expressed in the transcriptomic data, however they were not DE as gene transcripts at the time of sampling.

#### CSRP3 differential expression

The scaffold NW_019641951 contained six genes, including *CSRP3*. No differential expression between MFM and control WB was observed for *CSRP3* (*P* = 0.8; log_2_FC = 0.08) or any of the genes on this scaffold.

### Coding single nucleotide polymorphism analysis

A total of 72,365 coding single nucleotide polymorphisms (cSNP) were called for all 16 horses, of which 43.6% had a minor allele frequency > 0.1. There were 1208 variants that mapped to significant DEG and DEP. No significant coding SNPs associated with the MFM phenotype when comparing the 8 MFM and 8 control WB (Additional File 2, FDR ≤ 0.05). In the unplaced scaffold containing CSRP3, 236 coding SNPs were identified from the RNA-seq reads aligned to NW019641951. Of these, only 28 passed quality filtering with 11 mapping to CSRP3. No cSNP associated with the MFM phenotype.

### Co-inertia analysis

The co-inertia analysis (CIA) resulted in a global similarity between transcriptomics and proteomics (RV-coefficient) of 0.795. The cumulative proportion of variance estimated from the first two pairs of loading vectors were 0.806 (0.565 and 0.241, respectively). There were 71 proteins and 76 genes selected as the top divergent variables from the omics sample space. Five of the DEP (APOA1, HP, HCCS, CSRP3 and APOO) and four of the DEG (*CHAC1*, *HBB*, *ADAMDEC1* and *NR4A2*) were among the top selected in the CIA (Additional File 3).

### Enrichment analyses

#### Proteomics

GO biological process yielded one significant enrichment term, cytoskeletal organization (GO:0007010) containing 26 DE proteins. After background correction, there was no significant enrichment in either GO molecular function or GO cellular location (Additional File 4).

#### Transcriptomics

GO analysis for DEG after background correction revealed 15 significantly enriched GO biological process terms. The GO term with the lowest adjusted *P* value was response to ketone (GO:1901654, q = < 0.0001, 8 DE gene transcripts) (Additional File 5). Seven of the 8 response to ketone DE genes were also defined as response to steroid hormone. There were no significantly enriched GO terms for GO cellular location terms or GO molecular function (Additional File 4).

#### Amalgamated data

**Fig. 2:**
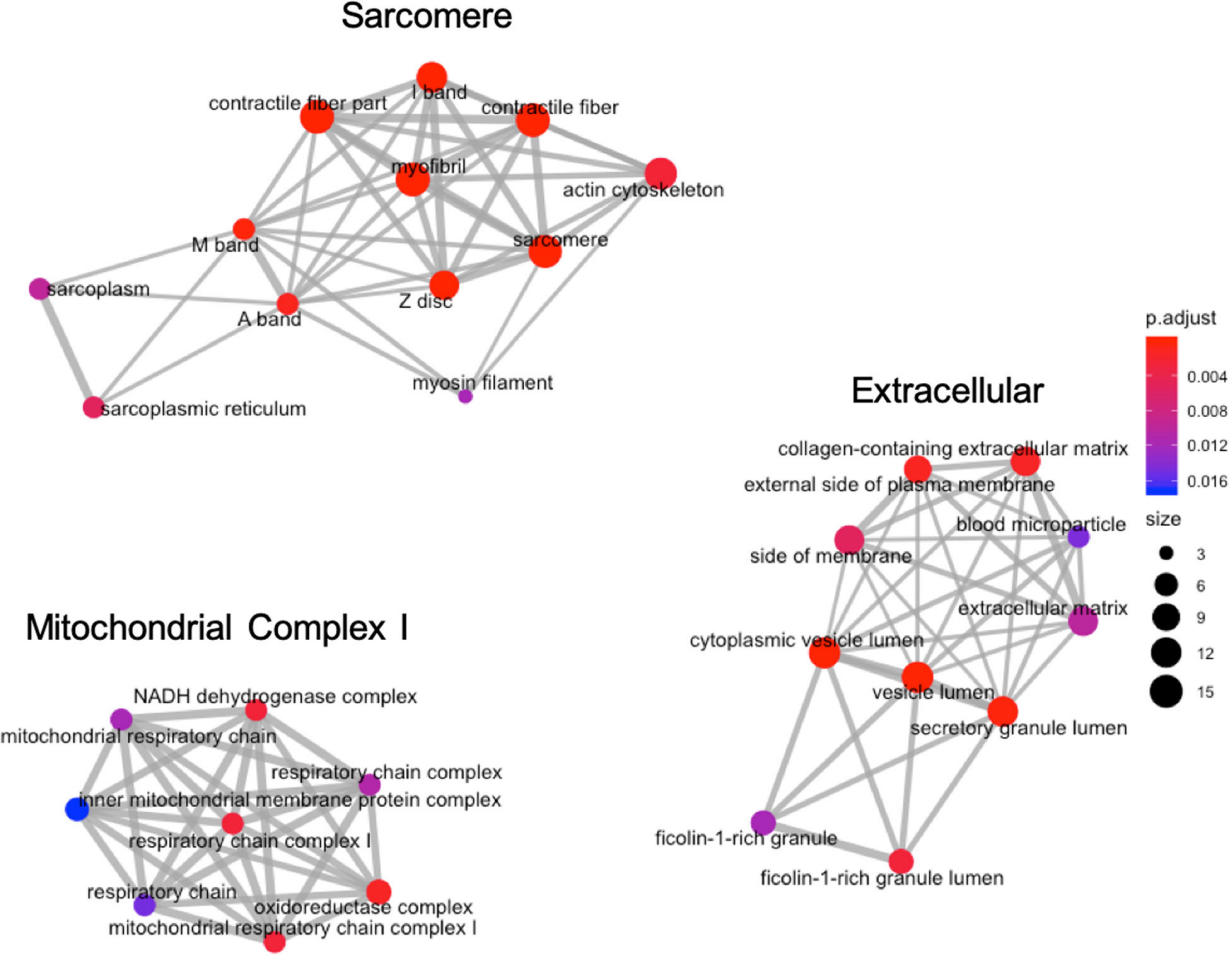
Enriched GO cellular location terms for DE gene transcripts merged with DE proteins in MFM WB. The size of the vertex indicates the number of DE target genes in that term. The color of the vertex indicates the adjusted *P* value and the edges (lines) connecting the vertices reflect DE target genes that were common between the GO terms

After merging both the DEP and the DEG gene IDs with a merged background correction, there was significant GO enrichment in biological process, molecular function, and cellular location terms. Many DEG and DEP appeared in multiple terms within their respective GO category (Additional Files 4, 5 and 6). Interestingly, the 45 significant GO terms for cellular locations had 3 distinct clusters that fell within 1) Z-disc and sarcomere structure, 2) complex I and the respiratory chain of mitochondria, and 3) extracellular matrix and vesicles (Fig. 2) (Additional File 4).

#### Co-inertia analysis

The top divergent genes and proteins selected from the co-inertia analysis were combined for pathway enrichment with merged background correction. Muscle system and circulatory system processes were significantly enriched for biological processes, lipoprotein particules for cellular component and ferroptosis for KEGG pathways (Additional File 7).

There were 126 significant GO biological terms and those that contained more than 10 gene IDs included: purine nucleotide metabolic process (7/39 terms), muscle cell development/ contraction/differentiation (6/39), ribonucleotide/ribophosphate metabolic process (4/39), nucleoside/tide metabolic process (3/39), cellular adhesion/regulation (3/39), response to inorganic/toxic substance (2/39), cofactor/precursor energy metabolism (2/39), heterocyclic or aromatic compound metabolism (2/39), apoptotic signaling (2/39), actin filament organization (2/39), blood circulation (2/39), response to oxidative stress/reactive oxygen species (2/39), nitrogen catabolic process (1/39), and protein post-translational modification (1/39) (Additional File 4).

There were 12 significant GO molecular functional terms included: NADH dehydrogenase/oxidoreductase activity (4/12), actinin/actin binding (3/12), protein lipid complex/binding (2/12), extracellular matrix/cell adhesion (2/12) and structural constituent of muscle (1/12) (Additional File 4).

Reactome pathway analysis of amalgamated data revealed 11 significantly enriched pathways. The pathway with the most DEP and DEG was metabolism of amino acids and derivatives (R-HSA-71291, q = 0.02). Similar to the GO analysis, there was overlap between pathways and DE gene IDs (Additional File 8), but metabolism of amino acids and derivatives (R-HSA-71291) and striated muscle contraction (R-HSA-390522, q = 0.07) were pathways that had no overlap. The remaining pathways were integrin signaling (R-HSA-9006921) with the largest amount of overlap in related pathways and complex I biogenesis (R-HSA-6799198) which shared DE genes with respiratory chain electron transport (R-HSA-611105) (Additional File 4).

### Amalgamated STRING analysis

After filtering, the STRING protein interaction network revealed 4 distinct clusters of protein interactions specific to the sarcomere, extracellular matrix, mitochondrial and ribosomal/translational activity (Additional Files 9 and 10).

### MFM electron microscopy

**Fig. 3:**
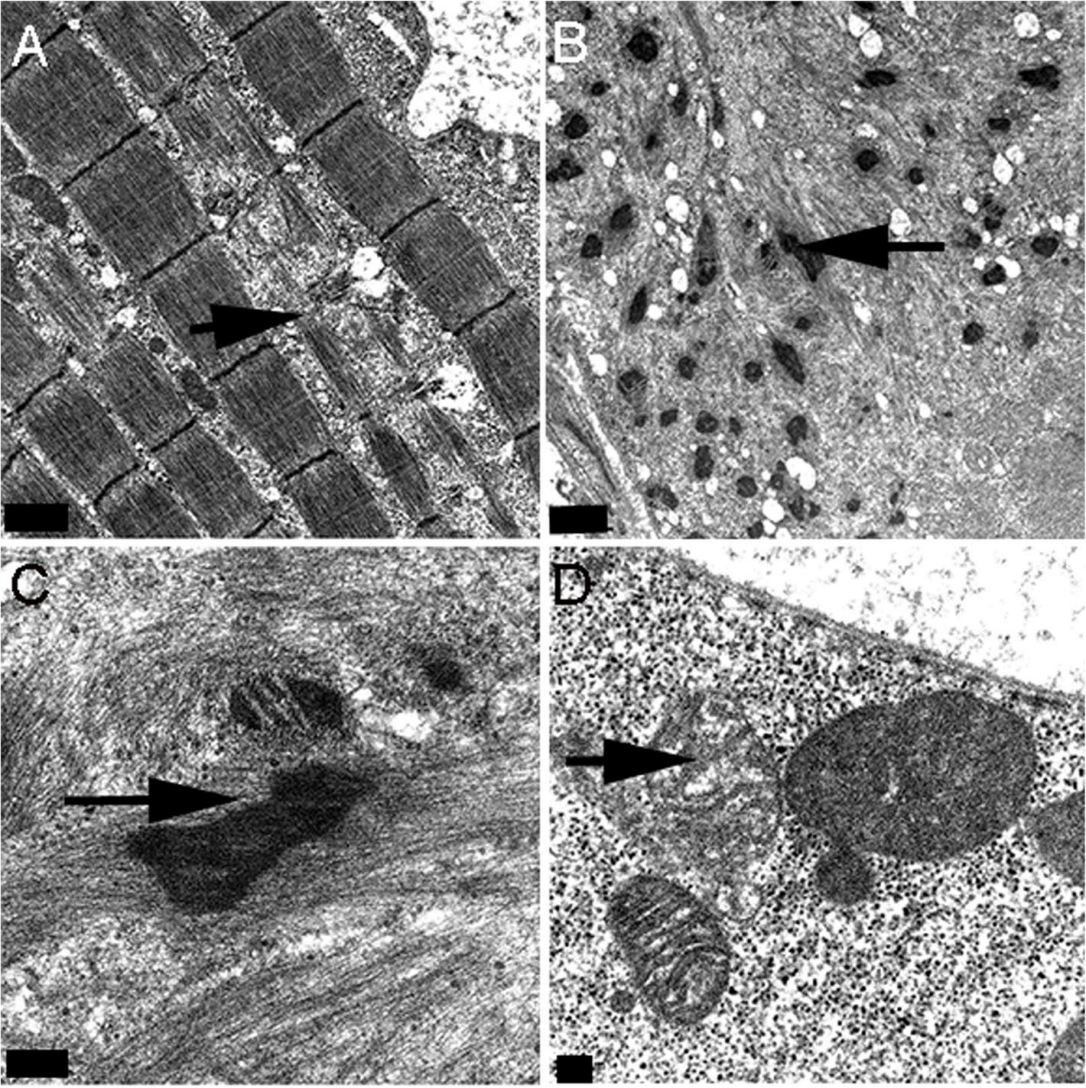
**A**. Normal appearing myofibrils adjacent to myofibrils with Z-disc disruption (arrow) and myofilament disarray in an MFM WB. 10 k. **B**. Marked myofilament disarray and ectopic accumulation of Z-disc material in an MFM WB. 10 k. **C**. Higher magnification of B. highlighting Z disc protein aggregation (arrow). 40 k. **D**. Mitochondria showing variability in size and cristae formation in an MFM WB. 27 k

Z-disc streaming and myofilament disarray were apparent in several regions of muscle fibers of 5 MFM WB examined with many other regions of myofibers having normal myofibril alignment (Fig. 3A). A few regions of myofibrils had severe myofibrillar disruption with notable ectopic accumulation of Z-disc material (Fig. 3B, C). Mitochondria appeared to have a normal appearance in many regions of the myofiber, however, subsarcolemmal areas contained mitochondria with pleomorphic shapes in some regions and other regions showed mitochondria varying in the density and arrangement of cristae (Fig. 3D).

### Immunofluorescent microscopy

**Fig. 4:**
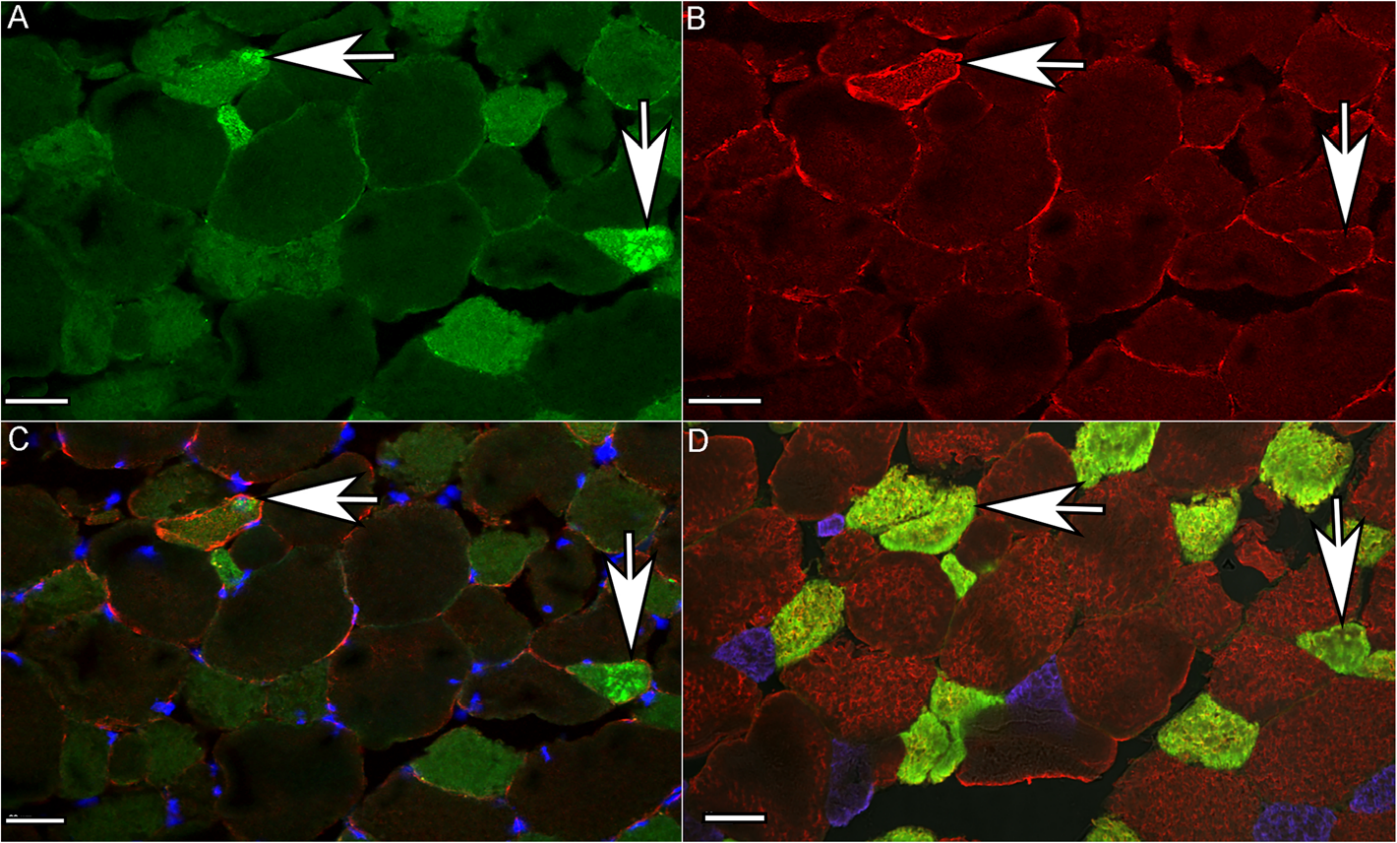
Immunofluorescent staining of cross-sections of muscle from an MFM horse. Horizontal and vertical arrows indicate the same fiber in all 4 images. Bar = 40 μm. **A**. Fibers with more intense CSRP3 staining or aggregates of CSRP3 correspond to type 2A fibers in D (vertical and horizontal arrows). **B**. Fibers with desmin aggregates corresponded to type 2A fibers in D (horizontal arrow). Some (vertical arrow) but not all fibers with desmin aggregates also had CSRP3 aggregates. **C**. Merged CSRP3 and desmin showing colocalization of CSRP3 with desmin aggregates (horizontal arrow) in some but not all fibers (vertical arrow). **D**. Fiber type stain showing type 1 fibers (blue), type 2A fibers (yellow) and type 2X fibers (brown)

**Fig. 5:**
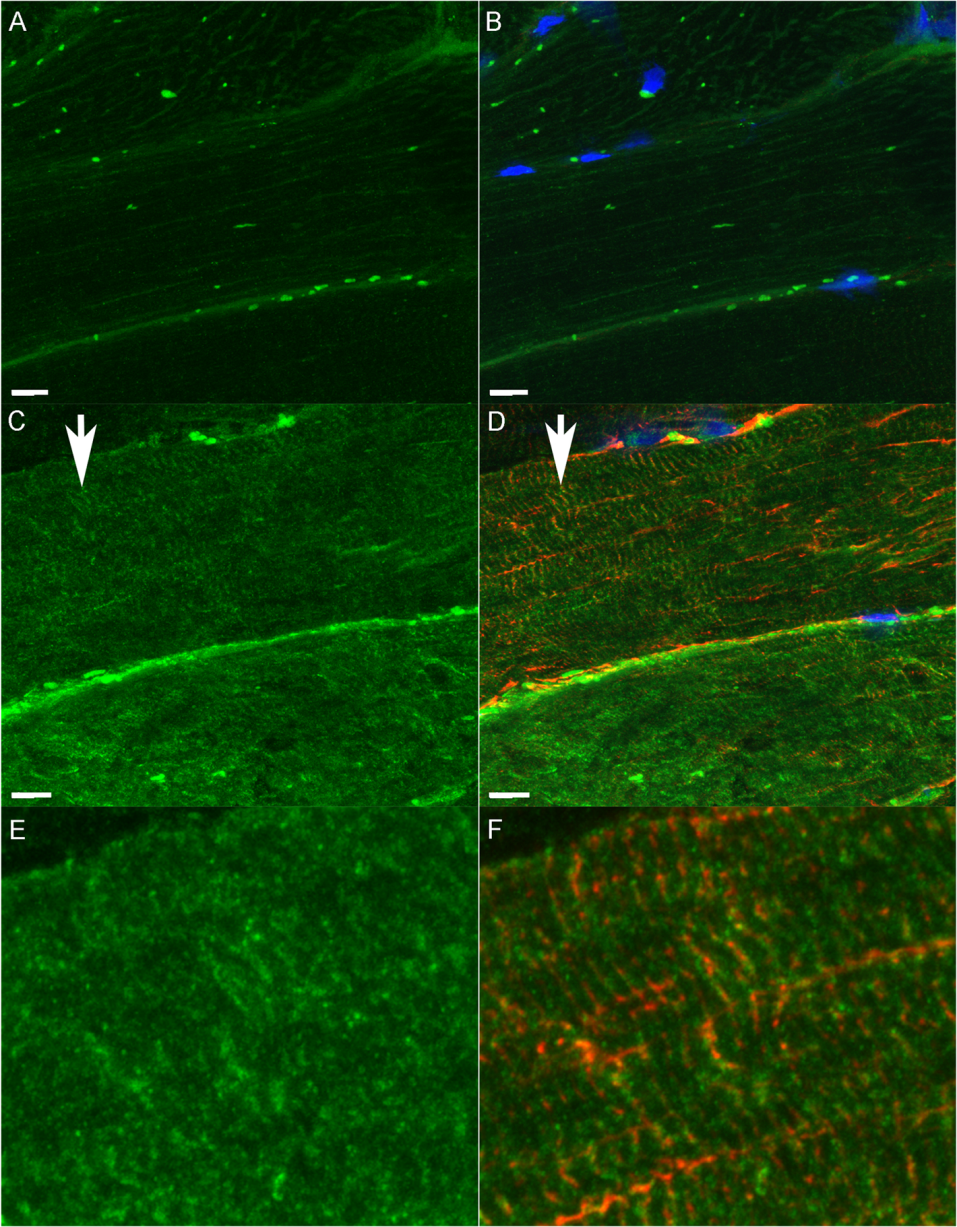
Immunofluorescent staining for CSRP3 and desmin in longitudinal sections of gluteal muscle. Bar = 10 μm. **A**. Section from a control horse stained with CSRP3 showing a normal pattern of staining. The fluorescent spheres appear to be red blood cells. **B**. Merged CSRP3 and desmin staining of the same section from a control horse. Nuclei are blue. **C**. Longitudinal section of an MFM horse stained with CSRP3 taken at the same intensity as the control horse in A. Some, but not all, fibers in the MFM horse had a striated appearance (vertical arrow). **D**. Merged CSRP3 and desmin staining of the same MFM section in C showing focal regions of the Z disc that had both CSRP3 and desmin staining (vertical arrow). **E**. Close up view of region under arrow in C showing some striation in the CSRP3 stain. **F**. Close up view of region under the arrow in D showing colocalization of CSRP3 with desmin in some areas

**Fig. 6:**
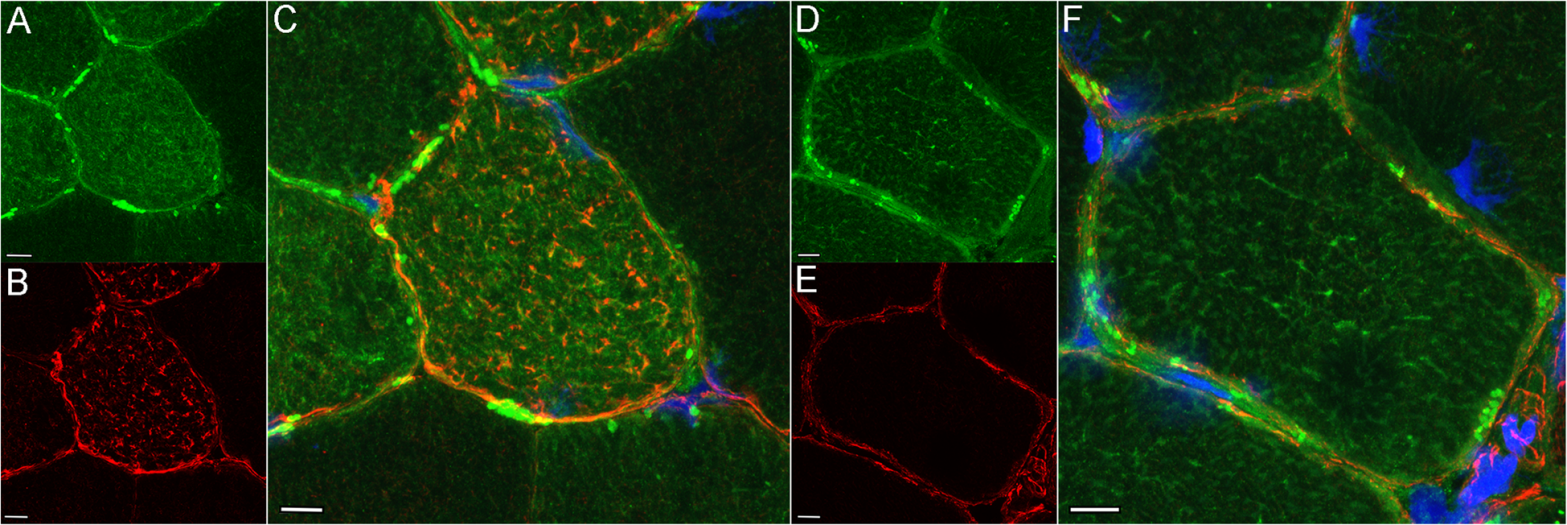
Immunofluorescent staining of cross-sections of a myofiber in gluteal muscle from an MFM WB and a control horse. Bar = 10 μm **A.** Disorganized pattern of CSRP3 staining in the myoplasm of an MFM horse compared to the control horse. **B.** Desmin aggregates in an MFM horse. **C.** Merged image showing colocalization of aggregates of desmin and CSRP3 (orange) in an MFM horse. Colocalization with CSRP3 occurred in some but not all fibers with desmin aggregates. **D.** Normal pattern of CSRP3 staining in a control horses taken at the same exposure as A. **E.** Normal pattern of desmin staining in a control horses taken at the same exposure as B. **F.** Merged desmin and CSRP3 stains in a control horse

Equine heart stained intensely for CSRP3 as a positive control, whereas sections incubated without the primary or secondary antibody had no background staining as did a tissue not expected to contain CSRP3 (equine liver) (Additional File 11). CSRP3 staining was evident in type 2A and occasionally type 2AX fibers (Fig. 4 A-D) of both control and MFM horses. CSRP3 staining had a striated appearance showing colocalization with desmin at the Z disk in some regions of MFM WB muscle fibers (Fig. 5 A-F). Muscle fibers with intense CSRP3 staining had a disrupted sarcoplasmic architecture compared to controls in MFM horses (Fig. 6 A-F). CSRP3 staining colocalized with desmin aggregates in some type 2A fibers (Fig. 6 A-C) (Additional File 12).

## Discussion

Myofibrillar myopathy can prematurely end an equine athlete’s career by causing exercise intolerance, muscle atrophy, myofibrillar disruption, and ectopic protein aggregation. Therefore, MFM has financial implications for horse owners, breeders, trainers, grooms, and riders. A naturally occurring equine model of MFM offers the opportunity to study the disease in depth while in a complex environment and also has comparative implications for human MFM. The present study is the first to utilize a combined multi-omic approach amalgamating proteomic and transcriptomic data to investigate biomarkers and unique molecular signatures of MFM in WB horses. Our amalgamated transcriptomic and proteomic enrichment analyses identified 3 distinct enriched cellular locations in MFM WB gluteal muscle which paralleled the ultrastructural skeletal muscle pathology. Forty-five enriched GO cellular location terms in our integrated analysis of MFM WB clustered to 1) the Z-disc and sarcomere, 2) complex I of the mitochondrial electron transport chain, and 3) the extracellular matrix. While there were additional enriched GO terms identified, the authors have prioritized discussion of the genes and proteins corresponding to the 3 cellular locations highlighted by the amalgamated proteomic and transcriptomic GO analysis in congruence with ultrastructural alterations observed in the electron micrographs. As more is discovered about MFM in WB, other pathways may have added significance in the pathogenesis of MFM.

Disruption of the Z-disc and ectopic Z-disc associated protein aggregation are hallmark histologic features of MFM in both humans and horses [8, 9, 11, 26]. Ultrastructural evaluation coupled with transcriptomic and proteomic enrichment analyses all underscored Z-disc pathology as a primary characteristic of MFM WB. Our proteomic analysis identified five α-actinin associated Z-disc proteins that had increased DEP in MFM WB. The co-inertia analysis identified nine additional actin cytoskeletal proteins and one gene as exhibiting divergent expression. Three of these proteins, SYNM, PDLIM3 (ALP) and CSRP3 (MLP) have well known functions in myocyte cytoarchitecture [27]. SYNM, an intermediate filament, anchors desmin to the Z-disc [28]. PDLIM3 colocalizes with α-actinin and enhances actin cross-linking [29]. CSRP3 interacts with telethonin, α-actinin, cofilin-2 and calcineurin at the Z- disc and has multiple proposed roles including maintenance of the cytoskeleton, myogenesis and autophagy [30, 31]. SYNM, CSRP3 and PDLIM are found in ectopic protein aggregates in human MFM together with desmin and numerous other proteins [28, 29, 32, 33]. Similarly, CSRP3 was also found to colocalize with some desmin aggregates in MFM WB.

Four of the DEP in MFM WB, CSRP3, PDLIM3, SYNPO2 (myopodin), SYNPOL2, are proteins integrally involved in the Z-disc’s role of sensing, integrating, and transducing biomechanical stress signals into signaling responses [27, 34, 35]. CSRP3 and SYNPO2 translocate from the cytoplasm to the nucleus in response to physical stretch, stress or strain where they impact gene transcription and myogenesis through interactions with transcription factors such as MyoD [36, 37]. The second most highly DEP, SMTNL1, also undergoes nuclear translocation with strong impacts on gene transcription [38, 39]. The integral role that these proteins have in gene transcription could indicate a potential disruption in adaptive responses to muscle strain or stress in MFM.

**Fig. 7:**
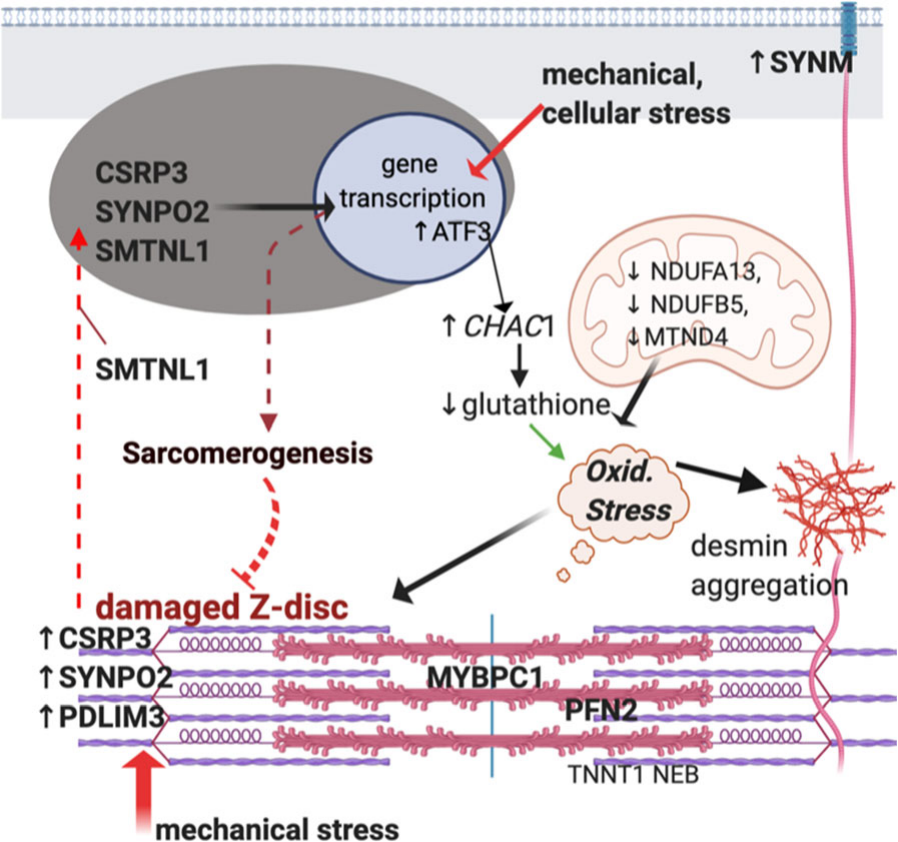
Proposed mechanism causing MFM WB based on DEG, DEP and enrichment analyses. Exercise combined with Z-disc instability in MFM WB induces an increase in PDLIM3, SYNPO3 and CSRP3 protein expression. Nuclear translocation of CSRP3, SYNPO2 and SMTNL1 with TF activation induces gene transcription designed to strengthen the sarcomere. A primary instability in the Z disc or a primary defect in mechanosignaling in MFM WB could lead to inadequate sarcomereogenesis and Z disc repair in MFM WB. A decrease in complex I mitochondrial subunits and enhanced glutathione degradation may be perturbing redox homeostasis with downstream effects of oxidative damage to proteins and aggregation of desmin [43]

Eccentric muscle contractions cause Z-disc damage and subsequent elevations in *CSRP3*, *PDLIM3* and *SYNPO2* expression [40, 41]. This type of exercise produces a compensatory adaptation in which the first insult from eccentric exercise triggers a signaling cascade that prevents damage when exposed to a second exercise bout [42]. The increase in CSRP3, SMTLN, PDLIM3 and SYNPO2 in WB MFM horses could represent an exaggerated adaptive signaling response designed to strengthen a pathologically weakened sarcomere. Alternatively, an ineffective mechano-signaling response by CSRP3, PDLIM3 and SYNPO2 could hinder the sarcomere’s ability to adapt and tolerate normal exercise-associated mechanical forces (Fig. 7) [43].

Among the four mechanosensing proteins, CSRP3 is considered a master regulator of muscle function and has aberrant primary expression patterns reported in human cardiomyopathy and secondary expression in skeletal muscle diseases [44, 45]. CSRP3 staining localized specifically to type 2A fibers which was the fiber type that also showed desmin aggregation in MFM WB. Silencing of CSRP3 results in the down-regulation of the expression of myogenic genes and the up-regulation of atrophy-related gene expression [45]. Mutations in CSRP3 have been associated with myofibrillar disarray and hypertrophic cardiomyopathy in humans [30, 46, 47], however, the coding sequence of CSRP3 in MFM WB did not contain any variants associated with the MFM phenotype. Thus, altered CSRP3 expression could be one of several proteins that play a role in the pathogenesis or response to the MFM WB phenotype.

In addition to the Z-disc, Complex I of the mitochondrial electron transfer system was a highlighted cellular location in MFM WB in the GO and STRING analyses. Seventeen mitochondrial proteins were DE (12 with ↓expression) in MFM WB relative to controls including 4 subunits of complex I. Furthermore, complex I biogenesis and oxidoreductase activity were enriched GO cellular and molecular function terms in the reactome and the amalgamated GO analysis in MFM WB. Decreased complex I expression could lead to altered mitochondrial function and cellular oxidative capabilities. Reduced complex I expression could, however, prove beneficial in circumstances where complex I is generating excessive reactive oxygen species.

Oxidative stress arises when reactive oxygen species, produced largely by complex I, overwhelm antioxidant capacity [48, 49]. Oxidative stress has been implicated as a key component of MFM in humans, Arabian MFM and may also play a role in MFM WB as suggested by the present enrichment analysis of DEG and DEP [8, 50–52]. The DEG with the largest fold change in MFM WB and among the top divergent genes in the CIA analysis was *CHAC1* (4.8 log_2_ fold change), which encodes an enzyme that degrades the thiol-based antioxidant glutathione. As a component of the unfolded protein response, activation of *CHAC1* serves as a cellular stress response designed to promote apoptosis [52–54]. *CHAC1* mRNA expression is promoted by activating transcription factor 3 and 4 (ATF3, ATF4) [53, 54]. *ATF3* was a significantly DEG (2.5 log_2_ FC) in MFM WB and *ATF4* although not DE was among the top divergent genes in CIA. Occurring after exercise, upregulation of *ATF3* regulates the expression of inflammatory cytokines and promotes molecular adaptations to endurance training [55]. Concurrent increases in *CHAC1* expression and enrichment of oxidative pathways could contribute to oxidative stress in MFM WB by increasing reactive oxygen species from complex I and enhancing degradation of the ubiquitous antioxidant glutathione in skeletal muscle. Although apoptosis is a beneficial survival response in mitotic tissues, it has limited benefits in post-mitotic cells such as muscle that are limited to satellite cell mediated repair [56]. Through its effects on protein structure, function, aggregation, and apoptosis, oxidative stress could further exacerbate sarcomere disarray and myofiber degeneration in MFM WB [51, 57, 58].

Interestingly, the extracellular matrix which plays a crucial role in mechanical force transduction, recruitment of satellite cells for repair, and muscle maintenance, was another enriched cellular location in the amalgamated GO and STRING analyses of MFM WB compared to control WB [59]. The extracellular matrix is connected to the sarcolemma through dystroglycan complexes and integrin mediated adhesion [60] Several of the DEG and DEP localized to the extracellular matrix (GO:0009897) in MFM WB, including increased expression of three fibrinogen subunits, thrombospondin and neural cell adhesion protein. Fibrinogen can be a driver of dystrophic skeletal muscle fibrosis [61]. The Reactome analysis highlighted integrin signaling pathways (R-HSA-9006921) [62, 63], which are activated by transduction of mechanical forces at the Z-disc to the sarcolemma through intermediate filaments and the cytoskeleton [64–68]. Upregulation of α7-integrin protein is believed to be a mechanism to reinforce muscle load-bearing structures and resist injury with repeated bouts of exercise [60]. Thus, upregulation of the extracellular matrix and proteins related to integrin signaling pathways could reflect a compensatory response of MFM WB muscle to prevent further sarcomere instability and damage.

The magnitude of log_2_ FC for all DEP was relatively small in our study, with CSRP3 showing the highest log_2_ FC at 0.74. Desmin itself was not a DEP, yet desmin aggregates in mature myofibers were used as the means for phenotyping horses. One potential explanation for the lack of desmin DE and low log_2_ FC in proteins in general could lie in the focal nature and fiber type specificity of MFM in WB horses. CSRP3 staining and desmin aggregates only occurred in type 2A and 2AX fibers within MFM-WB gluteal muscle and desmin aggregates only occurred in a small portion of these type 2A fibers in agreement with a previous study [9]. Analysis of many normal fibers combined with fewer affected fibers could potentially have diluted the magnitude of DEP. It is possible that more type 2A fibers are affected in MFM than those with desmin aggregates and that desmin aggregation in WB MFM represents a late secondary response to stabilize the Z-disc. Desmin aggregation can occur from a variety of causes beyond mutations in cytoskeletal or Z-disc proteins including dysfunction of ubiquitin-proteasome systems, aggresome formation, chaperone inhibited aggresome formation, abnormal p62 expression, and oxidative stress [51, 69–74].

Human MFM proteomic studies have used laser capture to compare the proteome of fibers with and without desmin aggregates within the same patients [32]. Significant differences in numerous proteins have been identified between affected and unaffected fibers using this approach with values reported as ratios of spectral intensities. The differences in reported spectral intensities and variation in study design make it difficult to compare the magnitude of protein expression between the human MFM studies and the present WB MFM study. Normalized log_2_ FC were assessed in a study of MFM Arabian horses [25] and only 3 proteins were DEP with approximately 3–4 log_2_ FC differences from control horses.

It was of note that the present study found little correlation between DEG and DEP. A lack of correlation between proteomic and transcriptomic expression profiles has been identified previously in other studies [75–77]. On the other hand the global similarity between our transcriptomics and proteomics was moderately high at 79%. Potential factors responsible for this disconnect include different half-lives of protein and mRNA, with half-lives of sarcomeric proteins being long (3 to 10 days), post-translational and transcriptional modifications, decreased proteasomal degradation, dysfunctional ubiquitination, negative feedback, and timing of cellular response [78–80]. The Z-disc is a dynamic structure with proteins exchanging out of and into the Z-band from cytoplasmic pools [81, 82]. In our study, horses were sampled at rest at only one time point which could have limited our ability to capture temporal relationships between gene and protein expression and impacts of exercise. Interstingly, the STRING protein interaction analysis also highlighted several DEP coorelating to ribosomal and protein translation which may warrant further investigation into the impact that those proteins play in this disease state. The lack of correspondence between DEG and DEP further emphasizes the importance of amalgamated analyses, as conducted in the present study, in elucidating molecular characteristics of diseases.

The present study was limited to transcript and protein expression analyses. Similar to genome first approaches, the individual assessment of transcriptomic or proteomic analyses provides a limited view into the cellular regulation and response to disease. However, the integration of two or more omic modalities has led to the expansion of personalized and precision medicine of both common and rare diseases ranging from monogenic to complex in origin [24]. Studies seeking to evaluate a more complete cellular perspective would ideally add genomic, epigenomic, and metabolomic profiling [22–24].

## Conclusion

An amalgamated transcriptomic and proteomic analysis identified 3 distinct cellular locations congruent with ultrastructural pathology in WB horses with MFM. The DEG and DEPs associated with these enriched locations have functions in Z-disc organization, mechanosignaling, oxidoreductase electron transport, and oxidative stress. It is possible that MFM in WB could arise from aberrant mechanosignaling proteins and oxidative stress with impacts on gene regulation, sarcomerogenesis, and complex I expression. The molecular signatures identified in the present study, may provide further insights into diagnostic biomarkers, potential treatments, and the pathophysiology of MFM in WB horses. Further studies, however, are needed to confirm dysregulation of specific pathways associated with MFM in WB horses.

## Materials and methods

### Sample collection

#### Case selection

The MFM WB horses included in the present study had a chronic history of poor performance and exercise intolerance not attributable to an orthopedic lameness. Control WB were matched to the same farm as MFM WB or were located within a 15-mile radius of an MFM WB except for one MFM WB that did not have a matching control. Control WB had no veterinary or owner documented history of exercise intolerance. One author (SJV) performed a physical examination of all horses.

#### Muscle biopsy and histologic analyses

Resting muscle biopsy samples were obtained by one author (SJV) from the gluteus medius muscle by percutaneous needle biopsy technique [83]. Horses had not undertaken strenuous exercise in at least the preceding 48 h. A portion of gluteal muscle was flash frozen in liquid nitrogen and stored at − 80 °C until analysis and if the sample was large enough, a portion was placed in formalin. Another portion of the sample was oriented in cross-section and frozen in isopentane chilled in liquid nitrogen. This sample underwent a battery of tinctorial and histochemical stains including adenosine triphosphate staining at pH 4.4 and desmin immunohistochemistry as previously described [9]. Formalin-fixed paraffin embed samples were stained with desmin. MFM horses had a minimum of > four (range 5–177) mature myofibers with desmin aggregates in muscle biopsies (Table 3). Control horses had no evidence of desmin aggregates or other histopathology. Muscle fiber types had been reported previously for the horses used in the present proteomic and transcriptomic analyses and there was no difference in muscle fiber type composition for horses (*P* = 0.21) (MFM WB type 1: 26% ± 11%, type 2: 74% ± 11%, control WB type 1: 32% ± 6%, type 2: 68% ± 6%) [13].

**Table 3:** MFM and control Warmblood (WB) horses used in this study, their sex (male, males castrate, female), age, number of muscle fibers with desmin aggregates, number of muscle fibers with desmin aggregates/ 4X high power filed (HPF), horses used in ATPase fiber typing, mRNA-sequencing, proteomic analysis electron microscopy (EM) and immunofluorescence microscopy (IF)

Horse	WB breed	Sex	Age (yrs)	Fibers with desmin agg.	Fibers with desmin agg. /HPF	ATPase Fiber typing	RNAseq	Proteomics	EM	IF
–	**MFM-WB**									
1	Danish WB	M	12	177	8	–	x	x	x	x
2	Dutch WB/ Thoroughbred	MC	10	61	5	–	x	–	–	x
3	Oldenburg	MC	8	15	5	x	x	x	–	–
4	Hanoverian/ Thoroughbred	F	18	17	5	x	x	x	x	x
5	Hanoverian/Trakehner	MC	10	40	5	x	x	–	–	–
6	Danish WB	F	16	49	12	x	x	x	–	x
7	Holsteiner	MC	14	5	1	x	x	x	–	–
8	Oldenburg	F	6	5	1	x	x	–	–	–
9	WB	M	8	36	5	–	–	–	–	x
10	Dutch WB	F	11	6	1	–	–	–	x	–
11	Holsteiner	MC	11	46	4	–	–	–	x	–
12	Dutch WB	F	9	12	3	–	–	–	x	–
**Control-WB**										
13	Oldenburg	M	18	0	0	x	x	–	–	–
14	Dutch WB/ Thoroughbred	MC	7	0	0	x	x	–	–	–
15	Westphalian	MC	13	0	0	x	x	–	–	x
16	Hanoverian	MC	13	0	0	x	x	x	–	–
17	Swedish	F	11	0	0	x	x	–	–	–
18	Holsteiner	F	19	0	0	x	x	x	–	–
19	Hanoverian	F	15	0	0	x	x	x	–	–
20	Belgian WB X	MC	10	0	0	x	x	x	–	–
21	Irish Sport Horse	MC	8	0	0	–	–	–	–	x
22	Dutch WB	MC	10	0	0	–	–	–	–	x

The research was approved by an institutional animal care and use committee at Michigan State University (IACUC AUF # 04/16–045-00) in compliance with the US National Research Council’s Guide for the Care and Use of Laboratory Animals, the US Public Health Service’s Policy on Humane Care and Use of Laboratory Animals, Guide for the Care and Use of Laboratory Animals, and Animal Research: Reporting of In Vivo Experiments guidelines.

### Proteomics

#### Horses

Muscle samples from five MFM WB (14.4 ± 3 yrs) and four control WB (13.8 ± 4.6 yrs) with one biological control replicate were analyzed on a 10-plex assay (Table 1).

#### Sample preparation, peptide fractionation and mass spectrometry

Protein was extracted from snap frozen gluteal muscle biopsies with a radioimmunoprecipitation lysis buffer [G Biosciences (https://ww.gbiosciences.com/)] and protease inhibitor with a ratio of 25 μl extraction buffer per mg of muscle, while on ice. Protein was then quantified with a bicinchoninic acid assay and pelleted. From each sample, 500 μg of protein was digested in trypsin with a Filter-Aided Sample Preparation protocol and spin ultrafiltration unit cutoff of 30,000 Da [84]. Reverse phase C18 SepPaks were used to de-salt the resulting peptides (Waters Corporation, Milford, MA) which were then dried by vacuum centrifugation. Peptide quantification was verified by colorimetric peptide concentration using 5 μL from each sample digest.

#### Isobaric labeling and gel fractionation

Samples containing 100 μg of peptide were re-suspended in 100 μL of 100 mM of TEAB and labeled with TMT10 reagents (Thermo Fisher Scientific, Waltham, MA) per manufacturer’s protocol. Labeling efficiency was tested by mass spectrometry with 5 μL from each sample. Equal sample proportions were mixed, de-salted, dried to a resulting volume of 2 μL and stored at -20C. Dried peptides were suspended in Agilent Offgel buffer to a volume of 1.5 mL and an Agilent 3100 OFFGEL Fractionator (Agilent, Santa Clara, CA) was used to fractionate the samples into 12 portions over a non-linear 3-10pH gradient per manufacturer’s instructions. Following electrophoresis, fractions were de-salted with C18 stageTips [85]. Samples were then dried to 2 μL via vacuum centrifugation and then frozen at -20C.

#### LC/MS/MS analysis

Samples were suspended in 2%ACN/0.1% Formic Acid to 20uL and an injection of 5uL was automatically made using a Thermo EASYnLC 1200 (Thermo Fisher Scientific, Waltham, MA) onto a Thermo Acclaim PepMap RSLC 0.1 mm × 20 mm C18 trapping column and washed for ~ 5 min with buffer A. Bound peptides were then eluted onto a Thermo Acclaim PepMap RSLC 0.075 mm × 250 mm C18 resolving column over 95 min with a gradient of 2%B to 32%B in 84 min, ramping to 100% B at 85 min and held at 100%B for the duration of the run (Buffer A = 99.9% Water/0.1% Formic Acid, Buffer B = 80% Acetonitrile/0.1% Formic Acid/19.9%H2O) at a constant flow rate of 300 nl/min. Column temperature was maintained at a constant temperature of 50 °C using and integrated column oven (PRSO-V1, Sonation GmbH, Biberach, Germany).

Eluted peptides were sprayed into a ThermoScientific Q-Exactive HF-X mass spectrometer (Thermo Fisher Scientific, Waltham, MA) using a FlexSpray spray ion source. Survey scans were taken in the Orbi trap (120,000 resolution, determined at m/z 200) and the top twenty ions in each survey scan are then subjected to automatic higher energy collision induced dissociation with fragment spectra acquired at 45,000 resolution. The resulting MS/MS spectra were converted to peak lists using Proteome Discoverer, v2.2 (Thermo Fisher Scientific, Waltham, MA) and searched against UniprotKB for all *Equus caballus* sequences. Results were appended with common laboratory contaminants using the Sequest HT search algorithm. The search output was then analyzed using Scaffold, v4.8.4 (Proteome Software, Inc., Portland, OR), to probabilistically validate protein identifications (FDR <  0.01). Quantification of reporter ion intensities is done using the Q + S module within Scaffold. The proteomic methodology used here has been previously validated and its use is standard protocol for the Michigan State Proteomics Core [86, 87].

#### Quantitative data analysis

Scaffold Q+ (version Scaffold 4.9.0, Proteome Software Inc., Portland, OR) was used to quantitate TMT Label Based Quantitation peptide and protein identifications. Peptide identifications were accepted if they could be established at greater than 95.0% probability by the Scaffold Local FDR algorithm. Protein identifications were accepted if they could be established at greater than 99.9% probability and contained at least 2 identified peptides. Protein probabilities were assigned by the Protein Prophet algorithm [88]. Proteins that contained similar peptides and could not be differentiated based on MS/MS analysis alone were grouped to satisfy the principles of parsimony. Proteins sharing significant peptide evidence were grouped into clusters. Channels were corrected by the matrix in all samples according to the algorithm described in i-Tracker [89]. Normalization was performed iteratively (across samples and spectra) on intensities, as described in Statistical Analysis of Relative Labeled Mass Spectrometry Data from Complex Samples Using ANOVA [90]. Medians were used for averaging. Spectra data were log-transformed, pruned of those matched to multiple proteins, and weighted by an adaptive intensity weighting algorithm. Of 108,665 spectra in the experiment at the given thresholds, 79,921 (74%) were included in quantitation. Differentially expressed proteins were determined by applying a permutation test and corrected by Benjamini-Hochberg (FDR ≤ 0.05, *P* ≤ 0.003). The mass spectrometry proteomics data have been deposited to the ProteomeXchange Consortium via the PRIDE [91] partner repository with the dataset identifier PXD019187 and 10.6019/PXD019187.

### Transcriptomics

#### Horses

Transcriptomic analysis was performed on muscle from eight MFM WB (mean age 13.6 ± 4 yrs) and eight control WB (13.9 ± 3 yrs) (Table 1).

#### RNA isolation

Total RNA was isolated from flash frozen *gluteus medius* samples as previously described [13]. Quantification and quality of RNA was assessed using a Qubit Fluorometer and RNA HS Assay Kit (Thermo Fisher Scientific, Waltham, MA) and RNA integrity (RIN) was determined using an Agilent 2100 Bioanalyzer and an Agilent RNA 6000 Pico Kit (Agilent Technologies, Santa Clara, CA). Samples with RIN > 7.0 were used for further analysis.

Library construction was performed with a strand-specific polyA capture protocol (TruSeq Stranded mRNA Library, (Illumina, San Diego, CA) and sequencing was performed in a 2x150bp paired end format using HiSeq 4000 SBS reagents for a target of 35–40 million reads for each sample. Base calling was done by Illumina Real Time Analysis (RTA) v2.7.7 and output of RTA was sorted and converted to FastQ format with Illumina Bcl2fastq v2.19.1 for analysis.

#### Assembly and mapping

All paired end RNA-seq reads were initially assessed for quality using FastQC [92] and MultiQC [93]. Reads with adapter sequence contamination were trimmed using Trimmomatic [94] and low quality reads (Q ≤ 30) were filtered with ConDeTri [95]. Filtered reads were mapped to the Equcab 3.0 reference genome (National Center of Biotechnology Information https://www.ncbi.nlm.nih.gov/assembly/GCF_002863925.1/) using Bowtie2 [96] indexes and the Tophat2 aligner [97]. SAMTools [98] was used to retain uniquely aligned reads (76%) for downstream analysis. The transcriptomes of each horse were assembled with Cufflinks [99] to create a unified annotation and quantify the expression of known and novel genes. HTSeq [100] was used to count the number of reads aligning to the annotated genes, retaining genes with expression observed across all horses in this study. Sequence data have been deposited in the NCBI Sequence Read Archive with BioProject number PRJNA603671 (accessions SRR10997329 to SRR10997344).

#### Differential expression and statistics

For the MFM WB and control WB, raw read counts per gene were normalized using the trimmed mean of M-values [101]. DE was determined by fitting a negative binomial generalized log-linear model per gene with diagnosis of MFM as coefficient of interest using EdgeR [102] and corrected for multiple testing with the Benjamini-Hochberg method with a false discovery rate ≤ 0.05.

#### Unplaced scaffold containing CSRP3

The most upregulated protein in MFM horses, cysteine and glycine-rich protein 3 (CSRP3), was located on the EquCab3.0 NW_019641951 scaffold that was discarded in the first quantitation. In order to include CSRP3 gene expression in our analysis, CSRP3 was assessed by aligning unmapped RNA-seq reads to NW_019641951 and DE analysis was then performed using the same negative binomial GLM.

### Coding single nucleotide polymorphism analysis

Coding single nucleotide polymorphisms were called from all significant DEG and DEP that had mapped gene IDs using SAMtools bcftools mpileup [103]. Variants that were called in 7 or more of the 8 horses per group (16 total MFM and control WB), had a PHRED score ≥ 30, and a minimum of 10 reads were retained for statistical analysis. Variant allele frequencies were compared between MFM and control WB using a Fisher’s exact test and a Benjamini-Hochberg test to account for multiple comparisons (*FDR <* 0.05). The cSNP analysis was also used to perform a genomic relationship matrix [104] which determined that the MFM horses were not more closely related than the control horses (maximum coefficient was 0.01).

### Enrichment analyses

#### Gene ontology analyses

Gene Ontology (GO) enrichment analyses was performed for DEG, DEP, and amalgamated DEG and DEP dataset. Significant DE gene symbols were translated to ENTREZ gene IDs in the R package org. Hs.eg.db using the human annotation [105–107] and were then analyzed and depicted with the default settings in clusterProfiler R package [108]. This provided pathway enrichment analysis for biological processes, cellular location, and molecular function.

#### Reactome pathway analyses

A reactome pathway analysis was performed for amalgamated DE genes and DE proteins. The converted ENTREZ gene IDs annotated to human were analyzed with the R package ReactomePA [109] and depicted with the default settings in clusterProfiler R package [108].

### STRING protein interaction network

A STRING protein interaction analysis was conducted on the amalgamated DEP and DEG using publically avalible software [110]. Physical networks were assessed based on confidence or strength of data supporting protein complexes. Active interaction sources included textmining, experiments, and databases. The minimum interaction score was set to the highest confidence (0.90).

### Transcriptome and proteome co-inertia analysis

A co-inertia analysis (CIA) was conducted to assess the correspondence between the collected transcriptomics and proteomics datasets. CIA is a multi-variate statistical method similar to canonical correlation analysis that aims to quantify the co-variability between two datasets. This correspondence is a global measure of variability known as the “co-intertia” parameter. To estimate this parameter we used the gene and proteins quantified for the five MFM and four control horses selected for proteomics. Log-cpm were estimated for the TMM normalized raw gene read counts and median protein spectral counts [111, 112]. CIA was performed on the centered and scaled log-cpm of genes and proteins with an identity matrix as positive weights for the samples and the Eluclidean metric of log-cpm as positive weights for the gene and proteins, repectively [113]. Pairs of optimal co-inertia loading vectors were estimated via eigenvalue decomposition as described [113]. The first two loading vectors of each omic-dataset were used to select the top 20 divergent genes and proteins from each quadrant and evaluated for pathway enrichment. Pathway enrichment analyses included GO, Reactome, and KEGG [105–107, 109, 114, 115].

### Electron microscopy

Electron microscopy (EM) had previously been performed on 2 MFM WB and one control-WB included in the transcriptomic and proteomic analyses [9]. EM was prospectively performed on 3 additional MFM WB to substantiate the ultrastructural changes with MFM (Horses 10–12, Table 1). Muscle was fixed in 2.5% glutaraldehyde in 0.1 mol/L sodium cacodylate buffer. Samples were dissected to approximately 5 mm^2^ oriented longitudinally and processed as previously described [8].

### Immunofluorescence microscopy

#### Horses

Frozen sections were evaluated for immunofluorescent (IF) microscopy from 5 MFM WB, 3 of which were used in the proteomic analysis, and 3 control horses. The use of horses not included in the proteomic analysis was dictating by availability of frozen muscle samples (Table 1).

#### IF staining

CSRP3 was selected for IF evaluation because it was the top differentially expressed protein and there was a paucity of literature on its expression in skeletal muscle. Frozen sections of equine heart served as positive controls, omission of primary and secondary antibodies served as negative controls. Desmin IF was performed using methods adapted from previously described immunohistochemistry in order to evaluate colocalization with CSRP3 at the Z-disc and colocalization in desmin aggregates [9].

Sections 10 μm thick were labeled with a human CSRP3 antibody targeting amino acids 77–122 which had high homology with the equine sequence (SC-166930; Santa Cruz Biotechnology, Inc., TX). Sections were thawed and fixed in ice-cold 100% acetone, blocked with 5% BSA, 2% goat serum in tris buffered saline with Tween 20 (TBST), and then incubated in fresh 1:100 CSRP3 antibody diluted in TBST. Sections were incubated with fresh secondary antibody, goat anti-mouse 488 (ab96879; AbCam; MA), diluted 1:100 in TBST, washed, blocked with 2% mouse serum TBST and incubated at *4* °C in fresh 1:100 desmin antibody (RB1178; Sigma-Aldrich; MO) diluted in TBST. After washes, sections were incubated with fresh secondary antibody, mouse anti-rabbit 555 (SC-516249; Santa Cruz Biotechnology, Inc., TX), washed and mounted in Vectashield with DAPI (H-1200; Vector Laboratories; CA). Sections were evaluated at 20X using a Zeiss Axiovert microscope. Confocal images were obtained using a Nikon C2 Confocal Laser Scanning Microscope configured on a Nikon Ni-U upright microscope with a 60x Plan Apochromat Lambda objective (NA 1.40). Images were acquired using the 60x objective (NA 1.40) at different confocal zoom magnifications using Nikon Elements software. The 561 nm diode laser was used for excitation and a 575-625 nm band pass emission filter was used to record the fluorescence. Fiber typing immunofluorescence was conducted as previously described [116].

## Supplementary Information

**Additional file 1.** The RNA-seq bioinformatic pipeline used.

**Additional file 2.** Coding Single Nucleotide Polymorphism analysis of mapped gene IDs from DEG and DEP failed to find any cSNPs associated with the MFM phenotype in 8 MFM and 8 control WB.

**Additional file 3.** Co-innertia analysis results. The top figure shows the sample space of the omic datasets including five MFM (red) and four control horses (black). The circles are the normalized score for each sample in the proteome and the arrow the normalized score for its transcriptome. The length of the line dividing the circle and the arrow is proportional to the divergence between the two datasets for that sample. The bottom two figures show the distribution of samples in the proteome (left) and transcriptome (right) space based on the first two estimated loading vectors with the top divergent variables shown in red.

**Additional file 4.** Gene Ontology (GO) and Reactome enrichment analyses for transcriptomics, proteomics and merged data.

**Additional file 5 **Eight GO biological function terms with the lowest *P* values for DE gene transcripts merged with DE proteins in MFM WB. The size of the bars indicates the number of DE genes/DE proteins in each GO term and the color of the bar reflects the adjusted *P* value.

**Additional file 6 **The GO molecular function terms for the merged DE gene transcripts and DE proteins. The color of the dots reflects the adjusted *P* value, the size of the dot reflects how many DE genes/proteins were included in that term, and the gene ratio indicates the number of DE genes/proteins in that term divided by the total significantly DE merged data count.

**Additional file 7.** Co-innertia analysis and enriched pathway data file [105–107, 109, 114, 115].

**Additional file 8 **The enriched reactome pathways of the merged DE gene transcripts and proteins. The size of the vertex indicates the number of DE target genes in that term. The color of the vertex indicates the adjusted *P* value and the edges connecting the vertices represents DE target genes that were common between the GO terms.

**Additional file 9.** STRING protein interaction map showing 4 clusters involving mitochondrial, sarcomere, ribosomal and extracellular matrix proteins.

**Additional file 10.** STRING protein interaction data file.

**Additional file 11.** Cross sections of control muscle samples. A. Horse heart stained for CSRP3 without primary antibody as a negative control. B. Horse heart stained for CSRP3 as a positive control with image obtained at the same intensity as A. C. MFM WB gluteal muscle stained for CSRP3 without primary antibody. D. MFM WB gluteal muscle stained for CSRP3 with image obtained at the same exposure as C.

**Additional file 12.** Immunofluorescent staining of cross-sections of gluteal muscle from an MFM horses. Arrows indicate the same fiber in all images. Bar = 40 μm A. Merged CSRP3 and desmin stains showing desmin aggregates in type 2A muscle fibers (arrow). B. Fiber typing of a serial section showing type 1 fibers (blue), type 2A fibers (yellow, arrow), type 2X fibers (brown) and type 2AX fibers (yellow-brown). C. Desmin stain showing aggregates of desmin in several type 2A fibers (arrow). D. CSRP3 staining of type 2A fibers (white arrow).

## Data Availability

The datasets supporting the conclusions of this article are available in the NCBI Sequence Read Archive with BioProject number PRJNA603671 (accessions SRR10997329 to SRR10997344; https://www.ncbi.nlm.nih.gov/bioproject/?term=PRJNA603671) and the ProteomeXchange Consortium via the PRIDE partner repository with the dataset identifier PXD019187 and 10.6019/PXD019187 (http://proteomecentral.proteomexchange.org/cgi/GetDataset?ID=PXD019187).

## Abbreviations

MFM: Myofibrillar myopathy
ACTB: b-actin
ATF3: Activating transcription factor 3
CHAC1: Glutathione specific gamma-glutamylcyclotransferase 1
CIA: Co-inertia analysis
cSNPs: Coding single nucleotide polymorphisms
CSRP3: Cysteine and glycine-rich protein 3
DDT: D-dopachrome decarboxylase
DE: Differential expression
DEG: Differentially expressed genes
DEP: Differentially expressed proteins
FC: Fold change
FDR: False discovery rate
GAPDH: Glyceraldehyde phosphate dehydrogenase
GO: Gene ontology
HBB: Hemoglobin subunit beta
IDs: Identifications
IF: Immunofluorescent
qRT-PCR: Quantitative real-time polymerase chain reaction
RIN: RNA integrity
RTA: Real Time Analysis
WB: Warmblood horses
