# Supplementary figures and images for: Integrated proteomic and transcriptomic profiling identifies aberrant gene and protein expression in the sarcomere, mitochondrial complex I, and the extracellular matrix in Warmblood horses with myofibrillar myopathy
Source: BMC Genomics. 2021 Jun 11;22:438. doi: 10.1186/s12864-021-07758-0 (PMC8194174; doi:10.1186/s12864-021-07758-0)

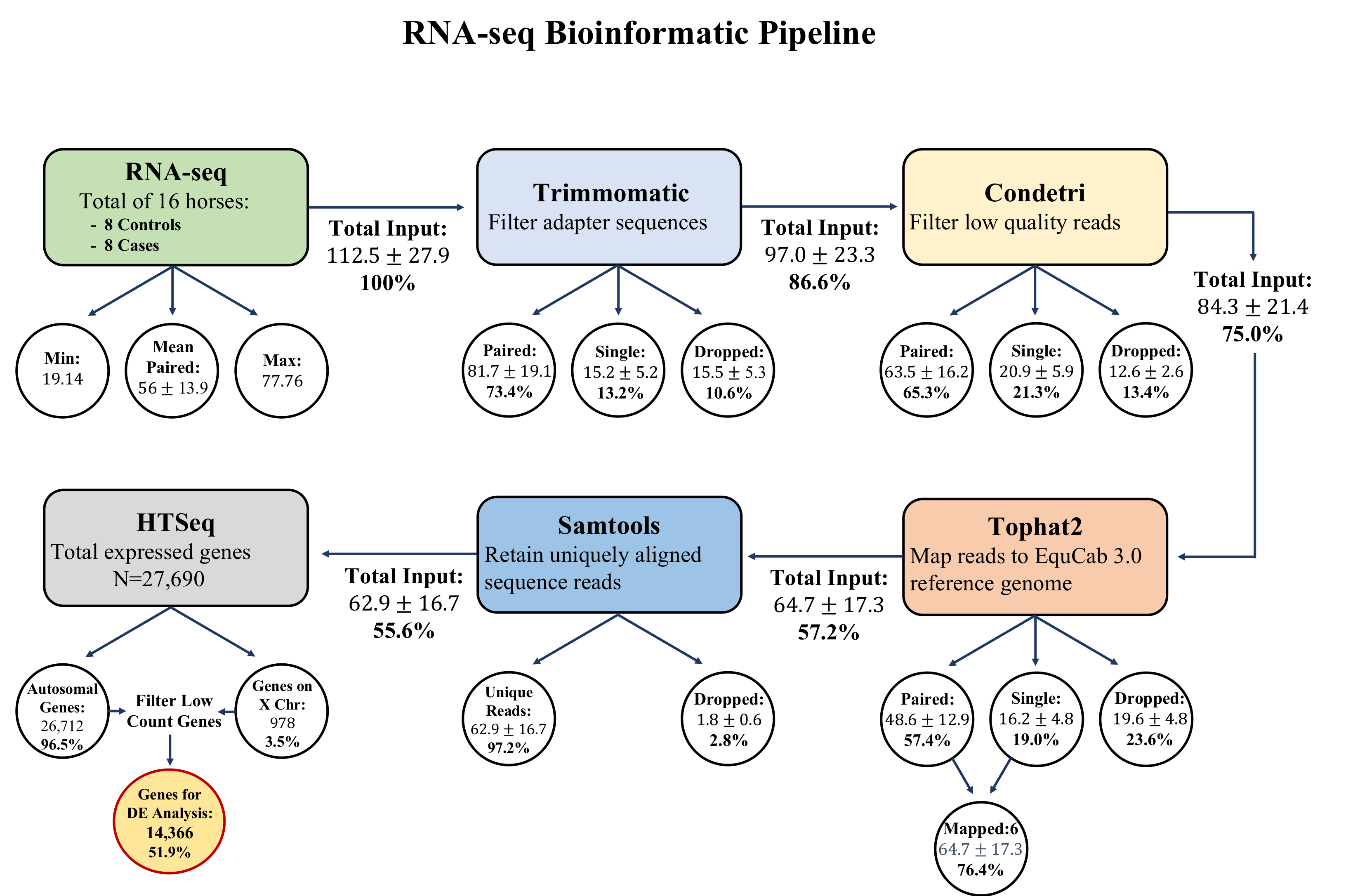

Supplement: Supplementary file 1 — Additional file 1. The RNA-seq bioinformatic pipeline used. [file 12864_2021_7758_MOESM1_ESM.png]

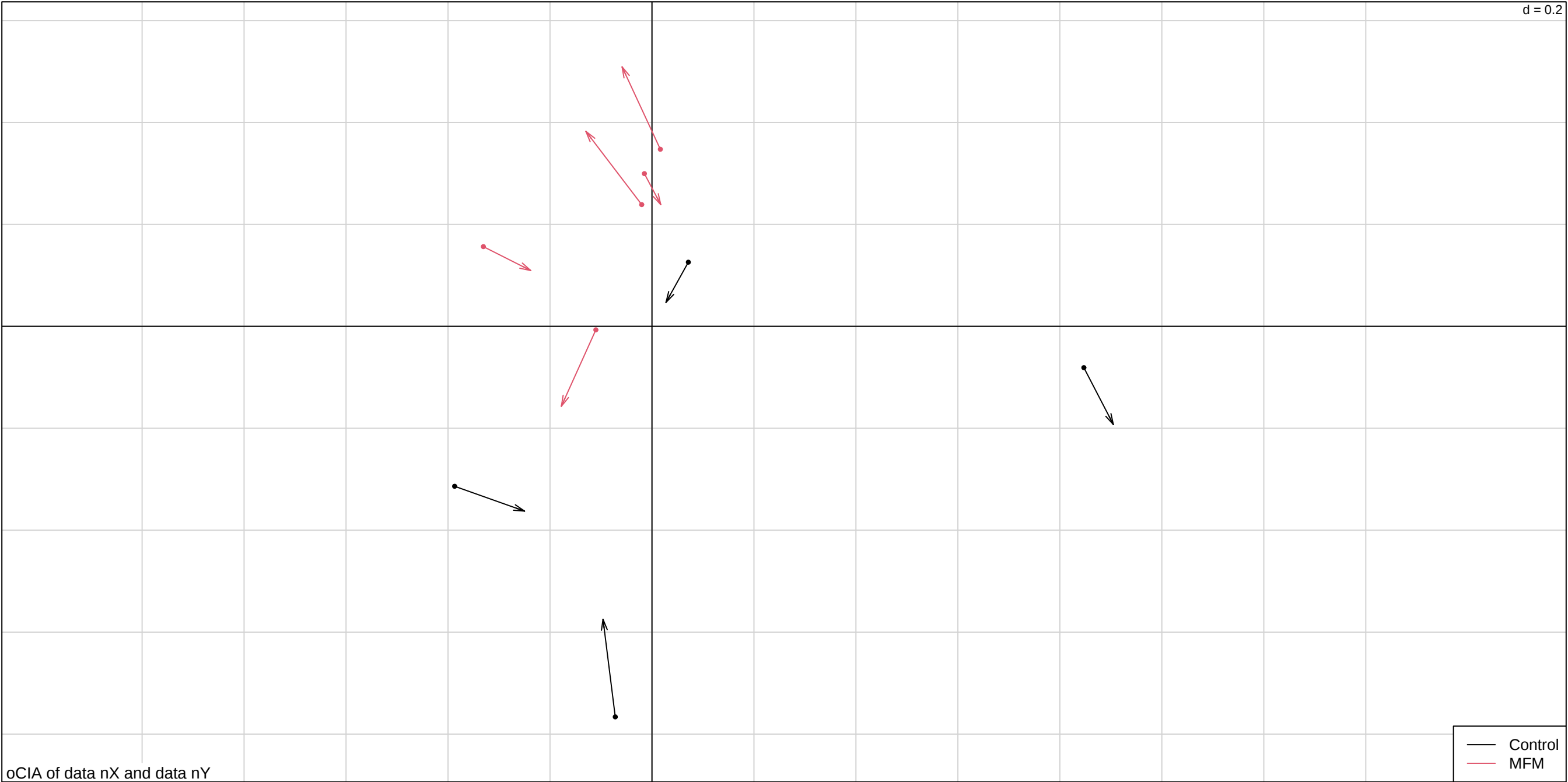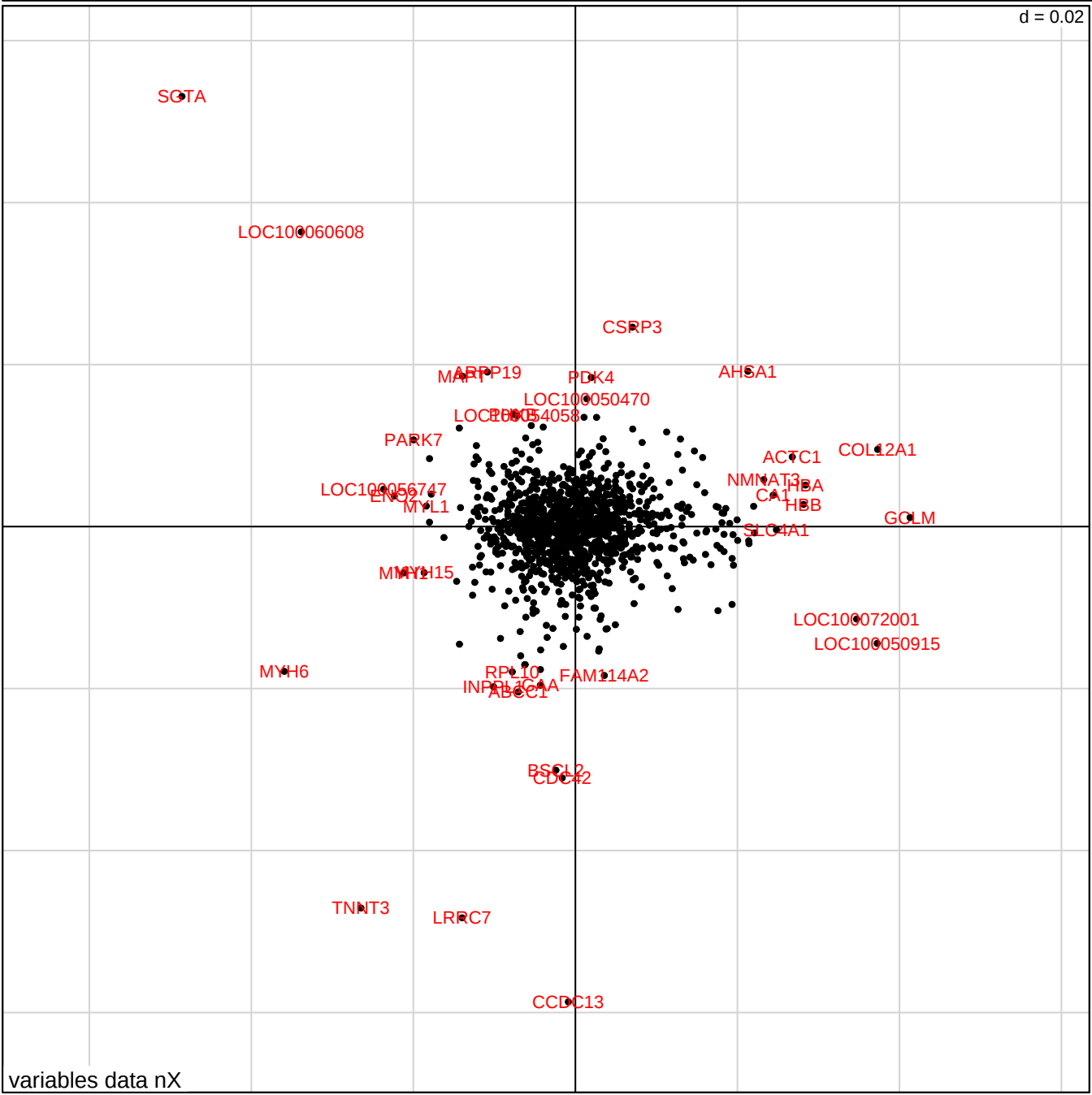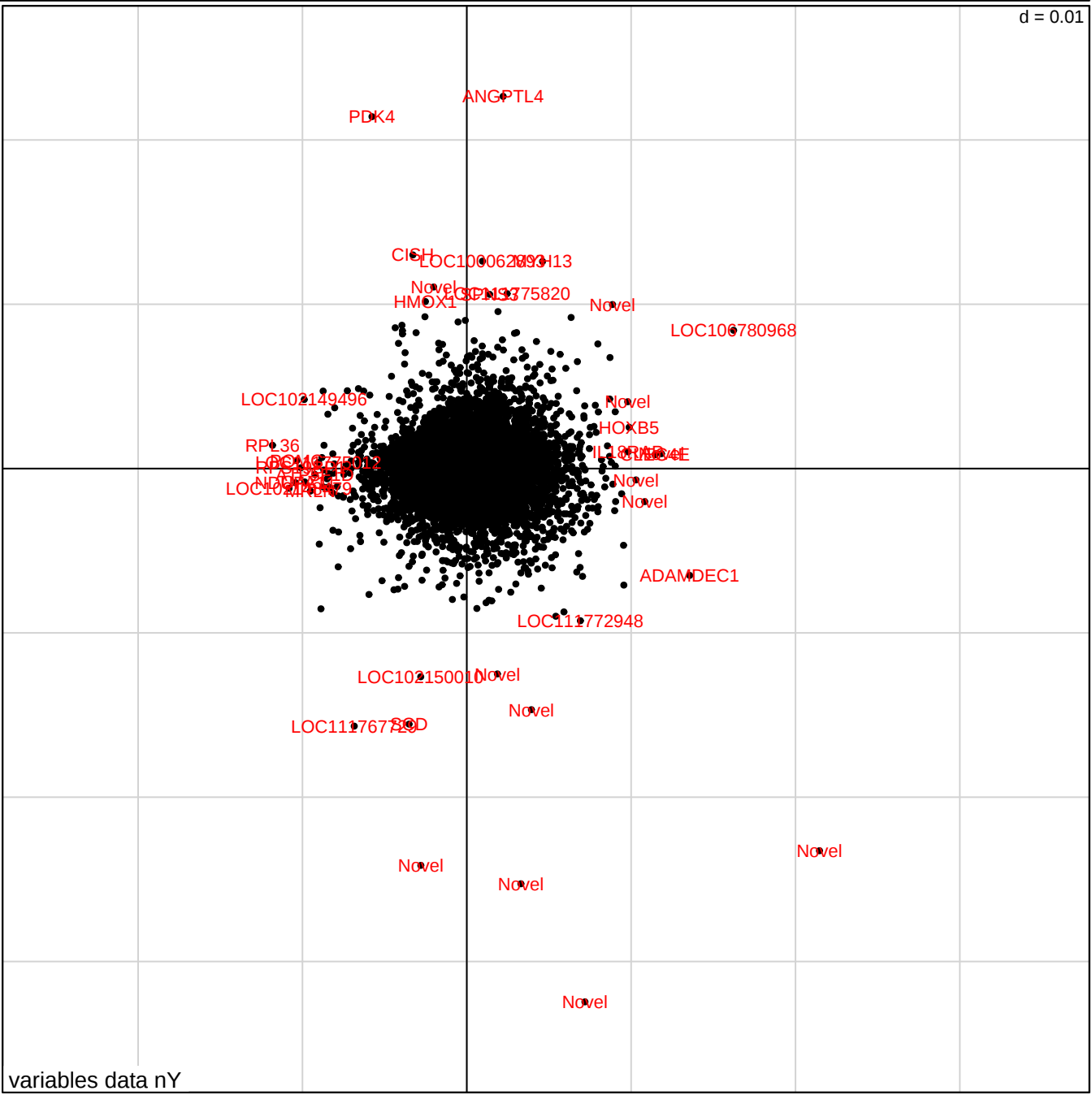

Supplement: Supplementary file 3 — Additional file 3. Co-innertia analysis results. The top figure shows the sample space of the omic datasets including five MFM (red) and four control horses (black). The circles are the normalized score for each sample in the proteome and the arrow the normalized score for its transcriptome. The length of the line dividing the circle and the arrow is proportional to the divergence between the two datasets for that sample. The bottom two figures show the distribution of samples in the proteome (left) and transcriptome (right) space based on the first two estimated loading vectors with the top divergent variables shown in red. [file 12864_2021_7758_MOESM3_ESM.pdf]

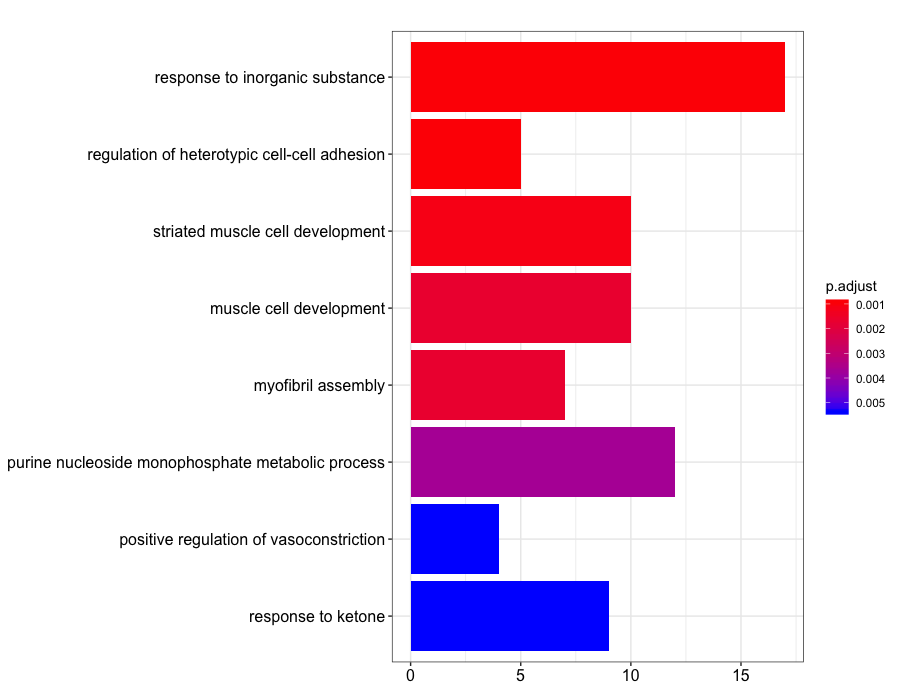

Supplement: Supplementary file 5 — Additional file 5 Eight GO biological function terms with the lowest P values for DE gene transcripts merged with DE proteins in MFM WB. The size of the bars indicates the number of DE genes/DE proteins in each GO term and the color of the bar reflects the adjusted P value. [file 12864_2021_7758_MOESM5_ESM.png]

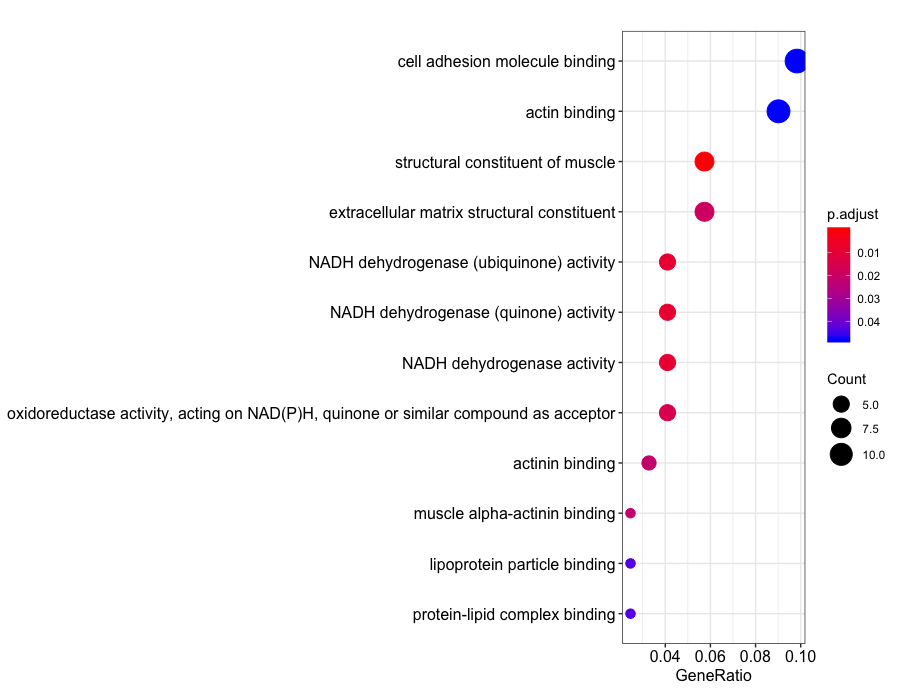

Supplement: Supplementary file 6 — Additional file 6 The GO molecular function terms for the merged DE gene transcripts and DE proteins. The color of the dots reflects the adjusted P value, the size of the dot reflects how many DE genes/proteins were included in that term, and the gene ratio indicates the number of DE genes/proteins in that term divided by the total significantly DE merged data count. [file 12864_2021_7758_MOESM6_ESM.png]

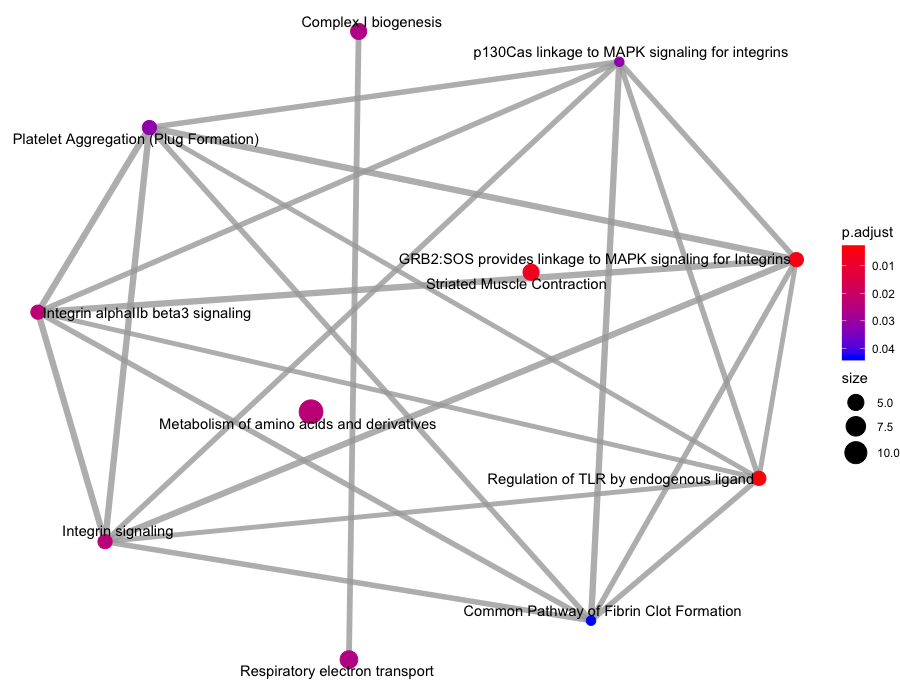

Supplement: Supplementary file 8 — Additional file 8 The enriched reactome pathways of the merged DE gene transcripts and proteins. The size of the vertex indicates the number of DE target genes in that term. The color of the vertex indicates the adjusted P value and the edges connecting the vertices represents DE target genes that were common between the GO terms. [file 12864_2021_7758_MOESM8_ESM.png]

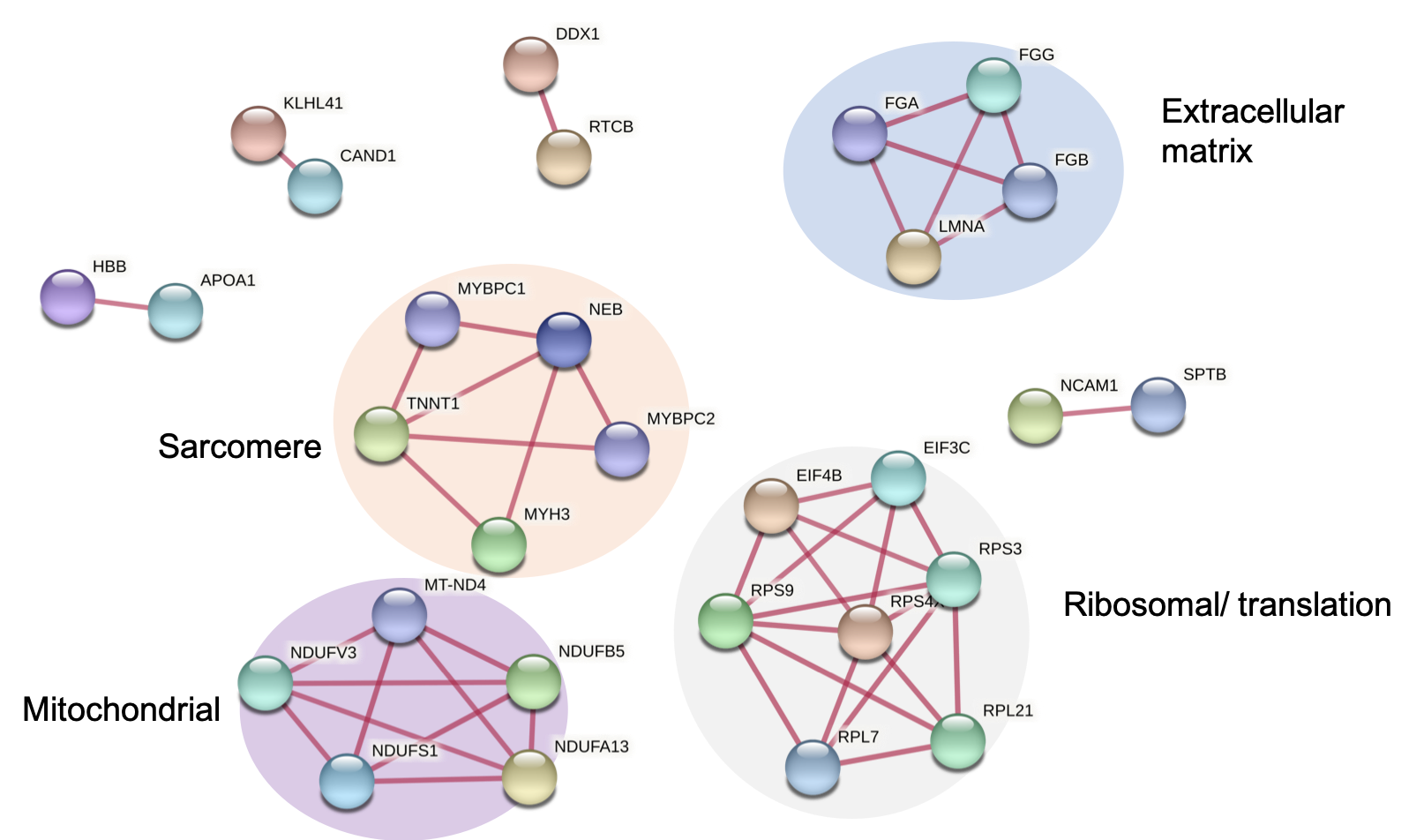

Supplement: Supplementary file 9 — Additional file 9. STRING protein interaction map showing 4 clusters involving mitochondrial, sarcomere, ribosomal and extracellular matrix proteins. [file 12864_2021_7758_MOESM9_ESM.png]

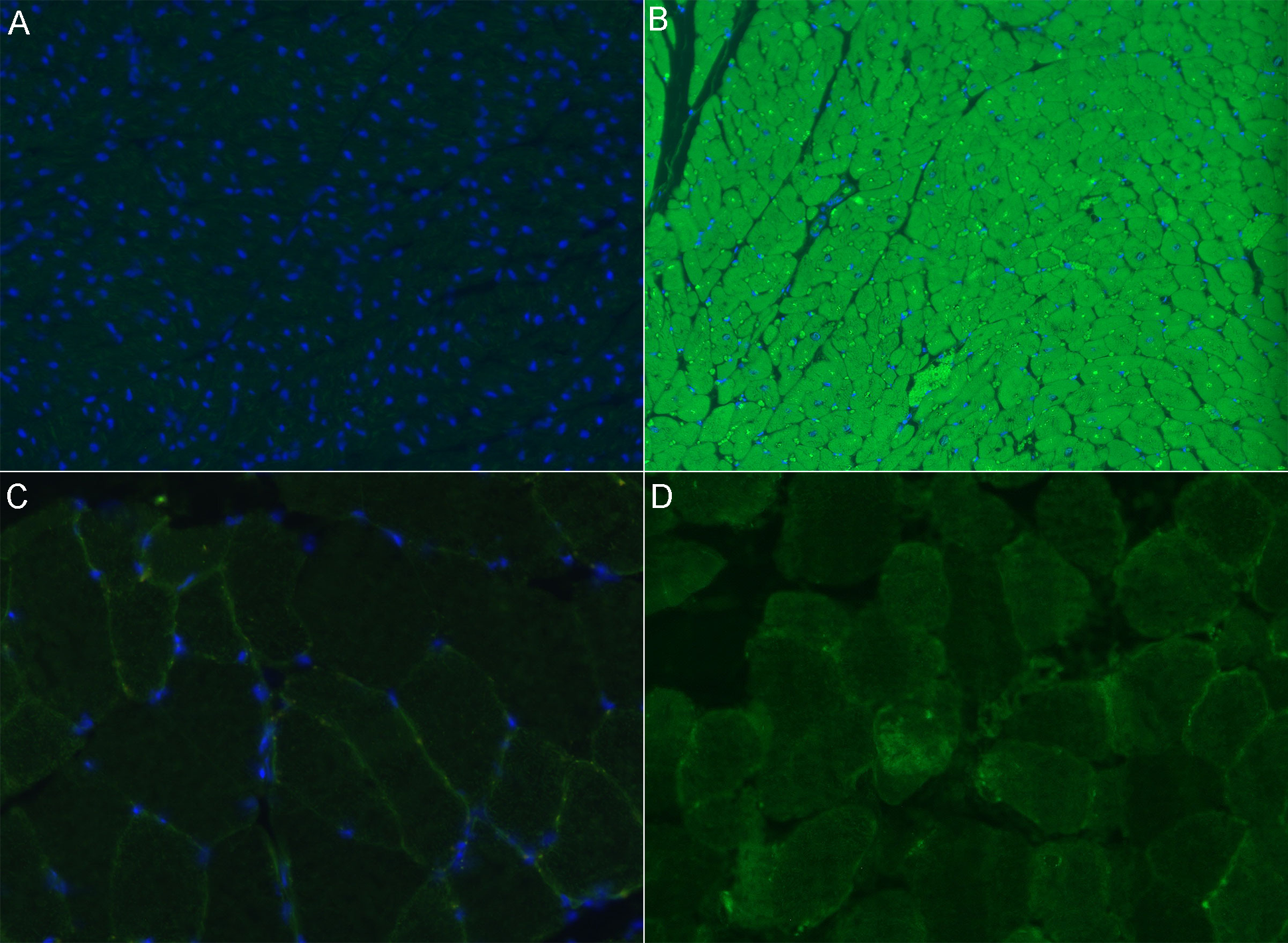

Supplement: Supplementary file 11 — Additional file 11. Cross sections of control muscle samples. A. Horse heart stained for CSRP3 without primary antibody as a negative control. B. Horse heart stained for CSRP3 as a positive control with image obtained at the same intensity as A. C. MFM WB gluteal muscle stained for CSRP3 without primary antibody. D. MFM WB gluteal muscle stained for CSRP3 with image obtained at the same exposure as C. [file 12864_2021_7758_MOESM11_ESM.tif]

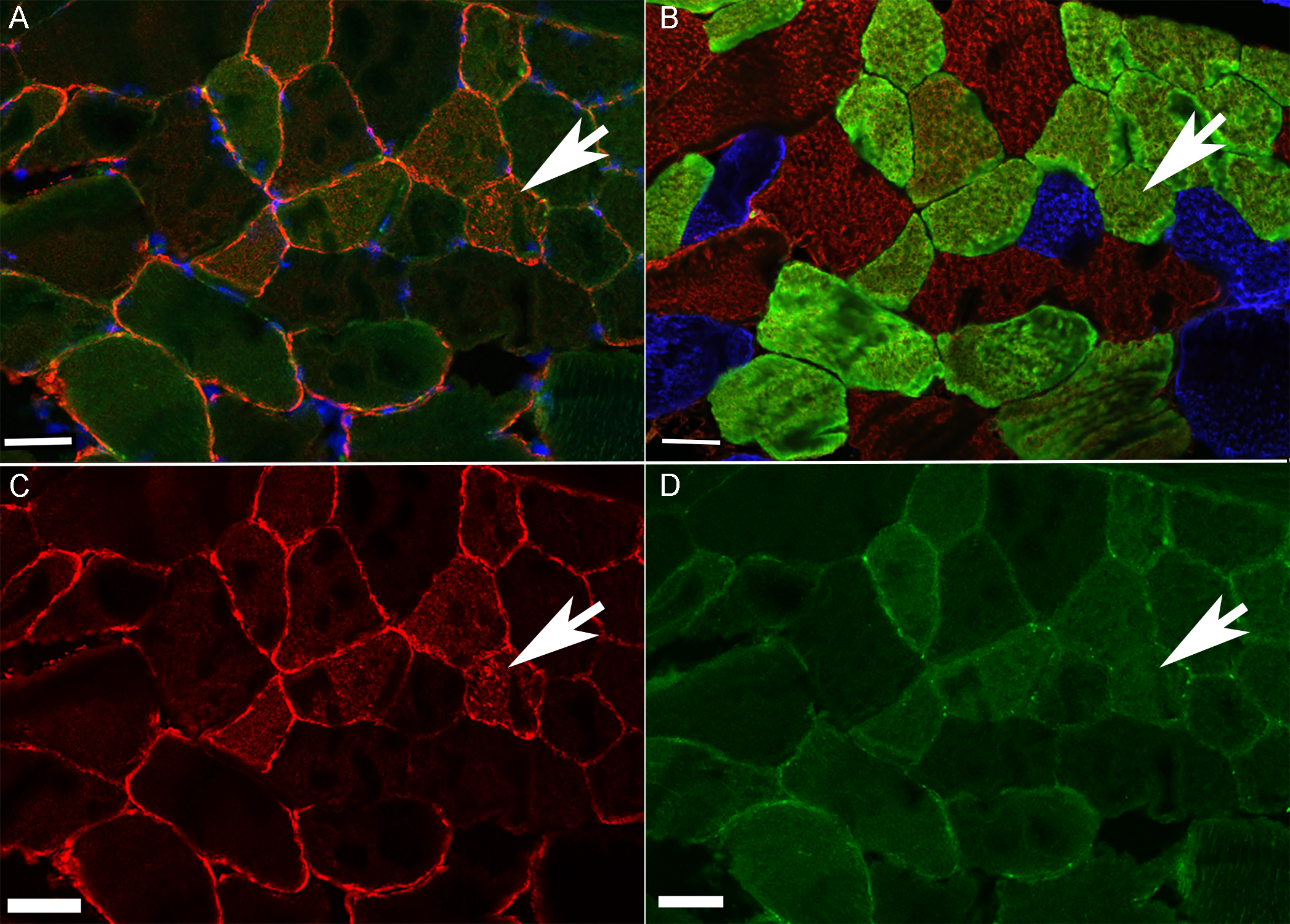

Supplement: Supplementary file 12 — Additional file 12. Immunofluorescent staining of cross-sections of gluteal muscle from an MFM horses. Arrows indicate the same fiber in all images. Bar = 40 μm A. Merged CSRP3 and desmin stains showing desmin aggregates in type 2A muscle fibers (arrow). B. Fiber typing of a serial section showing type 1 fibers (blue), type 2A fibers (yellow, arrow), type 2X fibers (brown) and type 2AX fibers (yellow-brown). C. Desmin stain showing aggregates of desmin in several type 2A fibers (arrow). D. CSRP3 staining of type 2A fibers (white arrow). [file 12864_2021_7758_MOESM12_ESM.tif]
